# Seasonal influenza vaccination in Kenya: an economic evaluation using dynamic transmission modelling

**DOI:** 10.1186/s12916-020-01687-7

**Published:** 2020-08-20

**Authors:** Jeanette Dawa, Gideon O. Emukule, Edwine Barasa, Marc Alain Widdowson, Omu Anzala, Edwin van Leeuwen, Marc Baguelin, Sandra S. Chaves, Rosalind M. Eggo

**Affiliations:** 1 KAVI-Institute of Clinical Research, College of Health Sciences, University of Nairobi, Nairobi, Kenya; 2 Washington State University Global Health Programs Kenya Office, Nairobi, Kenya; 3 Influenza Program, Centers for Disease Control and Prevention, Nairobi, Kenya; 4 Health Economics Research Unit, KEMRI Wellcome Trust Research Programme, Nairobi, Kenya; 5 Center for Tropical Medicine, Nuffield Department of Medicine, University of Oxford, Oxford, UK; 6 Division of Global Health Protection, Center for Global Health, Centers for Disease Control and Prevention, Nairobi, Kenya; 7 Division of Global Health Protection, Center for Global Health, Centers for Disease Control and Prevention, Atlanta, GA, USA; 8 Public Health England, London, UK; 9 London School of Hygiene & Tropical Medicine, London, UK; 10 Imperial College London, London, UK; 11 Influenza Division, National Center for Immunization and Respiratory Diseases, US Centers for Disease Control and Prevention, Atlanta, GA USA

**Keywords:** Dynamic transmission model, Economic evaluation, Cost-effectiveness, Influenza vaccine, Low- and middle-income countries, Vaccine timing, Vaccine target group

## Abstract

**Background:**

There is substantial burden of seasonal influenza in Kenya, which led the government to consider introducing a national influenza vaccination programme. Given the cost implications of a nationwide programme, local economic evaluation data are needed to inform policy on the design and benefits of influenza vaccination. We set out to estimate the cost-effectiveness of seasonal influenza vaccination in Kenya.

**Methods:**

We fitted an age-stratified dynamic transmission model to active surveillance data from patients with influenza from 2010 to 2018. Using a societal perspective, we developed a decision tree cost-effectiveness model and estimated the incremental cost-effectiveness ratio (ICER) per disability-adjusted life year (DALY) averted for three vaccine target groups: children 6–23 months (strategy I), 2–5 years (strategy II) and 6–14 years (strategy III) with either the Southern Hemisphere influenza vaccine (Strategy A) or Northern Hemisphere vaccine (Strategy B) or both (Strategy C: twice yearly vaccination campaigns, or Strategy D: year-round vaccination campaigns). We assessed cost-effectiveness by calculating incremental net monetary benefits (INMB) using a willingness-to-pay (WTP) threshold of 1–51% of the annual gross domestic product per capita ($17–$872).

**Results:**

The mean number of infections across all ages was 2–15 million per year. When vaccination was well timed to influenza activity, the annual mean ICER per DALY averted for vaccinating children 6–23 months ranged between $749 and $1385 for strategy IA, $442 and $1877 for strategy IB, $678 and $4106 for strategy IC and $1147 and $7933 for strategy ID. For children 2–5 years, it ranged between $945 and $1573 for strategy IIA, $563 and $1869 for strategy IIB, $662 and $4085 for strategy IIC, and $1169 and $7897 for strategy IID. For children 6–14 years, it ranged between $923 and $3116 for strategy IIIA, $1005 and $2223 for strategy IIIB, $883 and $4727 for strategy IIIC and $1467 and $6813 for strategy IIID. Overall, no vaccination strategy was cost-effective at the minimum ($17) and median ($445) WTP thresholds. Vaccinating children 6–23 months once a year had the highest mean INMB value at $872 (WTP threshold upper limit); however, this strategy had very low probability of the highest net benefit.

**Conclusion:**

Vaccinating children 6–23 months once a year was the most favourable vaccination option; however, the strategy is unlikely to be cost-effective given the current WTP thresholds.

## Background

Influenza is an important cause of respiratory illness in Kenya, especially in children under 5 and, in particular, young children under 2 [1, 2]. In 2016, the Kenya National Immunisation Technical Advisory Group (KENITAG) recommended annual seasonal influenza vaccination for children 6–23 months of age [3]. KENITAG further recommended pilot projects to generate additional local data to inform implementation of a nationwide influenza vaccination policy. In particular, KENITAG requested that local evidence be generated on influenza vaccine cost-effectiveness, because their recommendation largely relied on studies in non-African countries [4–6]. Given the cost implications of a nationwide programme, local economic evaluation data are needed to inform policy on the design and benefits of influenza vaccination in Kenya.

In countries with year-round influenza activity, the World Health Organization (WHO) recommends vaccination with the most recent influenza vaccine formulation before the primary peak in influenza activity [7, 8]. In Kenya, cases are observed year round [9], with an equal number of cases occurring during the Northern Hemisphere (NH) and Southern Hemisphere (SH) seasons [10]. There are no published influenza vaccine cost-effectiveness studies in Kenya. In other tropical settings with year-round influenza transmission, there is some quantification of the effect of elderly vaccination [11], but no evidence of the impact of vaccinating children.

Although evidence from intervention and observational studies on the indirect effects of influenza vaccination is limited [12], dynamic transmission models have proven useful to evaluate the effect of public health interventions targeted at infectious diseases, because they incorporate direct and indirect effects of vaccination [13, 14]. By doing so, it is possible to identify the optimal target group and coverage level for vaccination programmes, especially where the impact of herd immunity significantly alters disease incidence and outcomes [15].

Using a dynamic transmission model, researchers in the United Kingdom (UK) showed that expanding the influenza vaccination programme to include children 5–16 years of age would be the most efficient strategy in further reducing morbidity and mortality associated with influenza in their country [16]. We adapted this age-stratified transmission model to estimate the burden of disease associated with seasonal influenza from 2010 to 2018 in Kenya. Our objectives were to identify the most cost-effective target group and to estimate the ideal timing and vaccine formulation by comparing the incremental cost-effectiveness ratios (ICER) per disability-adjusted life year (DALY) averted for different vaccination scenarios. This information may assist policy makers in determining optimal seasonal influenza vaccination strategies.

## Methods

We obtained influenza surveillance data among patients hospitalised with severe acute respiratory illness (SARI) in Kenya from 2010 to 2018 and defined peaks in influenza activity. We fitted a transmission dynamic model to these epidemics by fitting the number of SARI cases, the number of tested samples and the number of influenza virus-positive samples, by week and age group (< 1, 1–5, 6–14, 15–19, 20–49 and ≥ 50 years of age). The virus-positive samples were categorised by influenza type and subtype: influenza B, influenza A H1N1pdm09 (A(H1N1)pdm09) and influenza A H3N2 (A(H3N2)). We set each influenza year from September to August the following year, except at the start of the study period because data were available from January 2010. Using epidemiological information, we then estimated the number of asymptomatic cases, symptomatic cases, deaths and DALYS due to influenza each year. Using health care utilisation data and costs of illness, we determined the number of health care utilisation events and costs of influenza each year. Thereafter, we modelled different influenza vaccination strategies and determined the ICER per DALY averted and incremental net monetary benefit (INMB) of each vaccination strategy.

### Influenza surveillance data

We used weekly numbers of patients hospitalised with SARI identified through the Kenyan national SARI surveillance system from 1 January 2010 to 31 December 2018 (Additional file 1). This system is run by the Ministry of Health in a few health facilities and supported by the Centers for Disease Control and Prevention (CDC), Kenya [9]. There are approximately 759 hospitals with inpatient capacity in the country, of which 50 have a bed capacity of ≥200 [17]. We used data from 5 of these larger health facilities: Siaya, Nyeri, Mombasa, Nakuru and Kakamega County Referral Hospitals where a well-established surveillance system was in place, and comprehensive data for the full study period was available (Additional file 2, section 1). We excluded data from circulation of influenza A(H1N1)pdm09 during the pandemic period (January 2010 to December 2011) to focus on seasonal epidemics, because the influenza A(H1N1)pdm09 pandemic did not present normal influenza activity in Kenya and was associated with higher level of severity than other circulating strains [18].

Hospitalised patients with SARI were included in the surveillance system if illness onset was acute (within 14 days from admission date) and they presented with fever (or history of fever) and cough. Nasopharyngeal (NP) and oropharyngeal (OP) samples were tested by real-time reverse transcription-polymerase chain reaction (rRT-PCR) from a random subset of these hospitalised patients [9]. Of the 24,480 cases identified through the SARI surveillance system, 80% were tested for influenza virus.

We defined hospital-specific catchment populations around each SARI surveillance site as the population within 10 kilometres (km) of the hospital. This was informed by a study in Kenya that showed 90% of children admitted with symptoms of a febrile illness, reside within 10 km of the health facility (Additional file 2, section 1) [19].

### Defining epidemics

Kenya has year-round influenza transmission. To allow fitting and simulation of vaccine impact in the model, we used the following activity-period decision rule. We identified periods of high influenza activity as ≥2 successive weeks where the proportion of subtype-specific test-positive cases was greater than the average weekly proportion during the entire study [20]. A period ended when there were ≥ 2 consecutive weeks where the proportion of subtype-specific positive cases was less than the weekly average. In addition, influenza-positive cases had to be observed in at least 3 of the 5 surveillance sites during the identified period so that periods identified were of widespread transmission. During model fitting, start and end dates were adjusted to centre the model peak to observed cases. If the posterior mean estimate of the net reproduction number at the start of the simulation was less than 1 (i.e. little evidence of sustained transmission), the period was excluded.

### Transmission model

We modified an age-stratified Susceptible-Exposed-Infectious-Recovered (SEIR) compartmental modelling framework previously used to inform influenza vaccination policy decision-making in the UK (Additional file 2, section 2) [16, 21]. The main differences between the UK model and Kenya model and their impact on the findings are summarised in the supplementary text (Additional file 2, section 7).

Mixing between age groups was governed by social contact survey data from Kenya collected from the coastal region [22]. The latent period was fixed at 0.8 days, and the infectious period at 1.8 days [16]. Probability of transmission and the fraction of the population susceptible varied each season, and the values were estimated during fitting. The SEIR compartments were stratified into vaccine-naïve and vaccinated populations. The model is available in the fluEvidenceSynthesis package in R [23, 24].

### Parameter inference

Parameters of the model were fitted to Kenyan surveillance data using Bayesian evidence synthesis [16]. For each season, we inferred the transmissibility of the virus, the susceptibility of 3 age groups (≤14 years, 15–49 years, ≥50 years), the initial number of infections, the number of infections introduced from outside Kenya, the probability of identifying an influenza-positive patient within the catchment population in each of 3 age groups (<1 years, 1–5 years, ≥6 years) and the number of subtype-specific influenza cases in the whole Kenyan population during each epidemic. We determined the age groupings for susceptibility and ascertainment based on comparisons of the best fit of the model to the observed data.

Where there was more than one circulating subtype during a season, we fitted the model separately to each subtype. We ran 500,000 Markov chain Monte Carlo (MCMC) iterations after a burn in of 200,000. We thinned the chain to 2% and all results are presented from 10,000 samples from the joint posterior distribution. Posterior mean values and 95% Bayesian credible intervals (CI) are given.

### Vaccination component

We considered 12 vaccination strategies using: (i) 3 age groups: 6–23 months, 2–5 years, 6–14 years; (ii) 4 vaccination timings: vaccination campaigns in April–June, October–December, or both (to coincide with NH or SH vaccine availability), and year-round vaccination (Table 1). Vaccination coverage levels varied by strategy. We assumed that year-round vaccination would achieve higher coverage levels than shorter campaigns due to longer availability of vaccine and that vaccination coverage of older children would be slightly higher than younger children, based on findings from a demonstration vaccination programme in Kenya [25]. For biannual vaccination, we assumed that individuals would only be vaccinated once per year and that in each vaccination period only individuals who had not been vaccinated in the preceding 12 months would receive vaccine. Vaccination was assumed to occur at a constant rate during the vaccination period.

**Table 1:** Vaccination scenarios modelled in three age groups and four vaccination timings

		Vaccination timing and uptake			
		A: Apr–Jun SH vaccine	B: Oct–Dec NH vaccine	C: Apr–Jun and Oct–Dec Both vaccines	D: Year-round Both vaccines
**Age group**	**I:** 6–23 months	30%	30%	45%	60%
	**II:** 2–5 years	35%	35%	50%	65%
	**III:** 6–14 years	40%	40%	55%	70%

Coverage values were set based on influenza vaccination studies in Kenya [25] and local consultation.

*NH* Northern Hemisphere,* SH* Southern Hemisphere

We assumed that the NH and SH vaccines provided “all-or-nothing protection”, i.e. for 80% vaccine effectiveness (VE), 80% of vaccinated people receive 100% protection from infection [26]. Protection lasted from the time of vaccination up to the end of the subtype specific influenza activity period. Vaccine protection was restricted to an epidemic and was not carried forward to future epidemics. We assumed that the NH vaccine provided protection against influenza activity that began between September of the same year and February of the next year and did not protect against influenza activity beginning between March and August. Similarly, the SH vaccine provided protection against influenza activity that began between March to August of the same year and did not provide protection against activity starting either earlier or later than these months.

Influenza vaccine effectiveness varies each year and differs across age groups. To simplify the model, we used subtype-specific published values of overall influenza VE to set a fixed value of VE in the model as either good (70%) or poor (42%) in all target age groups. If published VE was ≥50%, VE was modelled at 70% across all age groups; however, if VE was < 50%, VE was set at 42% in the model (Additional file 2, section 3). The choice of a fixed influenza VE value was informed by a systematic review and was validated in the original UK study [16].

### Economic evaluation

We used an economic evaluation decision tree to categorise infected individuals as asymptomatic, symptomatic with mild illness (upper respiratory tract (URT) infections) or symptomatic with severe illness (lower respiratory tract (LRT) infections) based on influenza challenge studies [27]. Those with mild illness were either seen at an outpatient clinic or were not medically attended, while patients with severe illness were either hospitalised or not. All those with mild illness were assumed to recover, while those with severe illness either recovered or died (Additional file 2, section 4).

**Fig. 1:**
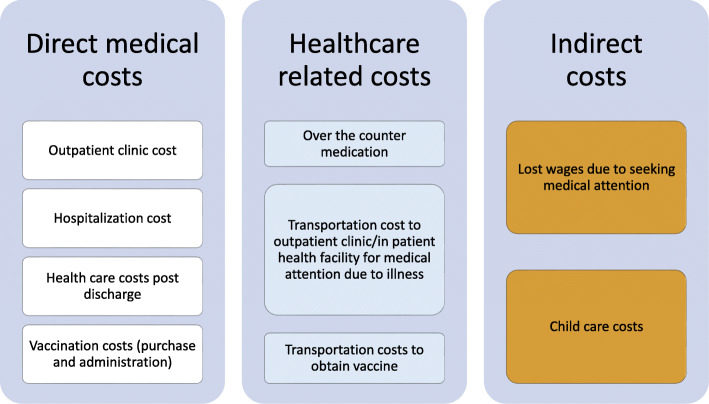
Summary of costs associated with influenza illness and vaccination. Shading of boxes: white = direct medical costs paid by government (presupposes a universal healthcare scheme with government as the main healthcare payer), blue = healthcare-related costs paid by individual, orange = indirect costs paid by individual

We calculated DALYs from disability weights of mild upper respiratory infection, moderate lower respiratory tract infection, severe lower respiratory tract infection and death [28] (Additional file 2, section 4). We estimated the proportion of cases that attended outpatient clinics or were hospitalised using representative South African influenza-specific healthcare utilisation data [29] (Table 2). To estimate costs from a societal perspective, we used an influenza costing study that described direct medical costs, healthcare-related costs, and indirect costs of influenza illness among patients with influenza attending health facilities in Kenya [30] (Fig. 1) (Table 3).

**Table 2:** Values for disease states and heath utilisation rates used in economic model. Mean and 95% confidence interval (CI) or proportions are given

Item	Measure	Value	Distribution	Reference
**Disease states**				
Proportion of influenza cases that develop any clinical symptoms	Mean (95% CI)	0.669 (0.583–0.745)	Normal	[27]
Proportion of influenza cases that develop upper respiratory tract symptoms/mild illness	Mean (95% CI)	0.588 (0.455–0.708)	Normal	[27]
Proportion of influenza cases that develop lower respiratory tract symptoms/severe illness	Mean (95% CI)	0.210 (0.140–0.303)	Normal	[27]
Proportion of influenza cases with severe illness that die while hospitalised				
*< 1 year*	Mean (95% CI)	0.0274 (0–0.0616)	Truncated normal	Influenza SARI surveillance dataset (2010–2018)
*1–5 years*	Mean (95% CI)	0.0091 (0–0.0322)		
*6–14 years*	Mean (95% CI)	0.0108 (0–0.0902)		
*15–20 years*	Mean (95% CI)	0 (0–0.1116)		
*20–49 years*	Mean (95% CI)	0.0331 (0–0.0818)		
*≥ 50 years*	Mean (95% CI)	0.1818 (0.0909–0.3080)		
*All ages*	Mean (95% CI)	0.0200 (0.0035–0.0373)		
Proportion of deaths due to a respiratory illness that occur in a health facility				
*< 1 year*	Mean (95% CI)	0.2794 (0.2451–0.3140)	Normal	Siaya health demographic and surveillance site dataset (2010–2016)
*1–5 years*	Mean (95% CI)	0.2899 (0.2471–0.3349)		
*6–14 years*	Mean (95% CI)	0.4361 (0.3534–0.5278)		
*15–20 years*	Mean (95% CI)	0.5250 (0.3750–0.6795)		
*20–49 years*	Mean (95% CI)	0.5067 (0.4626–0.5525)		
*≥ 50 years*	Mean (95% CI)	0.2715 (0.2421–0.3012)		
*All ages*	Mean (95% CI)	0.3287 (0.3106–0.3474)		
**Health care utilisation events**				
Proportion of symptomatic influenza cases who attend outpatient clinic				
*0–5 years*	Mean (95% CI)	0.475 (0.39–0.60)	Normal	[29]
*6–12 years*	Mean (95% CI)	0.118 (0.09–0.17)		
*13–17 years*	Mean (95% CI)	0.088 (0.06–0.13)		
*18–24 years*	Mean (95% CI)	0.035 (0.02–0.08)		
*25–44 years*	Mean (95% CI)	0.034 (0.02–0.07)		
*45–64 years*	Mean (95% CI)	0.027 (0.01–0.05)		
*≥ 65 years*	Mean (95% CI)	0.036 (0.02–0.07)		
Proportion of symptomatic influenza cases who are hospitalised				
*0–5 years*	Mean (95% CI)	0.0102 (0.0089–0.0117)	Normal	[29]
*6–12 years*	Mean (95% CI)	0.0007 (0.0006–0.0010)		
*13–17 years*	Mean (95% CI)	0.0006 (0.0004–0.0011)		
*18–24 years*	Mean (95% CI)	0.0008 (0.0006–0.0010)		
*25–44 years*	Mean (95% CI)	0.0021 (0.0018–0.0024)		
*45–64 years*	Mean (95% CI)	0.0026 (0.0020–0.0033)		
*≥ 65 years*	Mean (95% CI)	0.0033 (0.0025–0.0044)		
Proportion of outpatient influenza cases who purchased medication prior to clinic visit	Proportion	0.718	Fixed value	[30]
Proportion of hospitalised influenza cases who sought care after discharge from hospital	Proportion	0.105	Fixed value	[30]
Proportion of non-medically attended influenza cases where household members missed work due to illness*	Proportion	Not known	–	–
Proportion of outpatient influenza cases where household members missed work due to illness	Proportion	0.518	Fixed value	[30]
Proportion of hospitalised influenza cases where household members missed work due to illness	Proportion	0.848	Fixed value	[30]
Proportion of non-medically attended influenza cases where household members paid for childcare during illness*	Proportion	Not known	–	–
Proportion of outpatient influenza cases where household members paid for childcare during illness	Proportion	0.18	Fixed value	[30]
Proportion of hospitalised influenza cases where household members paid for childcare during illness	Proportion	0.29	Fixed value	[30]

*These items were not included in the model as the values were unknown and difficult to estimate in the case of non-medically attended illness

**Table 3:** Cost of influenza-associated illness in US dollars showing year of valuation

Type of cost	Measure	Value in USD	Year	Distribution	Source
**Direct medical costs**					
Facility based medical costs among influenza cases attending outpatient clinic	Mean (SD)	4.34 (1.30)	2014	Normal	[30]
Facility based medical costs among hospitalised influenza cases	Mean (SD)	59.19 (59.39)	2014	Normal	[30]
Health care costs after discharge among hospitalised influenza cases who sought care after discharge	Mean (SD)	3.28 (6.19)		Normal	[30]
Influenza vaccine purchase costs per dose (varied in sensitivity analysis)	Fixed	3	2018	Fixed value	Assumption
Vaccine administration cost per dose					
Supply chain cost per dose from national level to the health facility	Mean	0.43	2012	Fixed value	[31]
Provision of immunisation services at the health facility	Mean (SD)	1.0 (0.72)	2012	Normal	[31]
**Health care related costs**					
Transportation costs among influenza cases attending outpatient clinic	Mean (SD)	0.40 (0.87)	2014	Normal	[30]
Transportation costs among hospitalised influenza cases	Mean (SD)	5.03 (8.32)	2014	Normal	[30]
Transportation costs to receive vaccine at health facility	Man (SD)	0.20 (0.435)	2014	Normal	Assumption*
Health care costs prior to outpatient visit among influenza cases who purchased medication before the outpatient visit	Mean (SD)	1.39 (3.90)	2014	Normal	[30]
**Indirect costs**					
Lost wages among influenza cases not seeking formal health care for mild illness	Fixed	0	–	Fixed value	Assumption
Lost wages among influenza cases attending outpatient visit who report that household members missed work	Mean (SD)	12.84 (27.17)	2014	Normal	[30]
Lost wages among hospitalised influenza cases who report that household members missed work	Mean (SD)	42.02 (41.54)	2014	Normal	[30]
Lost wages among those not hospitalised with severe influenza illness	–	Not known	–	–	–
Childcare costs among influenza cases attending outpatient clinic who report household members paid for childcare	Mean (SD)	0.07 (0.57)	2014	Normal	[30]
Childcare costs among hospitalised influenza cases who report household members paid for childcare	Mean (SD)	0.11 (0.75)	2014	Normal	[30]
Childcare among those not hospitalised for severe influenza illness	–	Not known	–	–	–

*For this cost no data existed and an assumption was made that the cost would be half of the transportation costs for outpatient care

In the case where no data was available for costs incurred by non-medically attended cases, these costs were not included in the model

*SD* standard deviation

Vaccine administrative costs were obtained from a vaccine delivery costing study in Kenya and Tanzania (Table 3) [31]. We set vaccine purchase price at $3 US dollars (USD) per dose for a multi-dose vial, which was considered a reasonable price based on available market prices for the trivalent inactivated vaccine, if obtained through a negotiated agreement for low- and middle-income countries (LMICs). We tested the sensitivity of our results to this cost. Vaccine wastage was assumed to be 15% [32]. Costs from before 2018 were adjusted to 2018 USD values using the annual Kenya gross domestic product (GDP) deflator values.

We calculated annual ICERs per DALY averted for all 12 strategies compared to no vaccination (base scenario), as influenza vaccination was negligible in Kenya during the study period. We time discounted DALYs by 3% [33]. Average annual ICERs per DALY averted were calculated for each vaccine strategy. A cost saving output resulted in an increase in benefit and overall decrease in total costs incurred. For each strategy, we calculated the probability that it had the highest INMB at willingness-to-pay (WTP) thresholds of 1–51% of the 2018 Kenya GDP per capita (i.e. between $17 and $872 per DALY averted), and used the results to construct cost-effectiveness acceptability curves [34]. We then constructed cost-effectiveness acceptability frontiers depicting the highest probability for the most optimal strategy (i.e. the strategy with highest average INMB) [35].

In sensitivity analysis, we calculated DALYs with and without social weighting and time discounting [33]. Social weighting placed greater value on life lost from 9 to 56 years of age. We tested the impact of changing the vaccine purchase price to $1.5, $3.0, $6.0 and $10.0 per dose. Finally, we tested the impact of maintaining the same vaccine coverage across all age groups, i.e. 30% coverage for once yearly campaigns, 45% coverage for twice yearly campaigns and 60% for year-round vaccination.

### Ethics statement

Permission to undertake secondary data analysis of de-identified SARI surveillance data collected from patients admitted at county referral hospitals, was obtained from the Kenyatta National Hospital – University of Nairobi Ethics Review Committee (P18/01/2017).

## Results

### Periods of high influenza activity

**Fig. 2:**
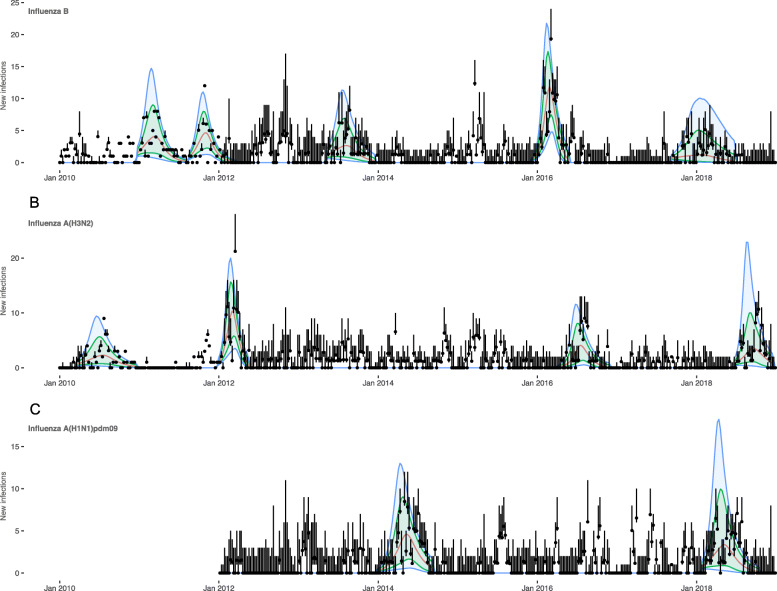
Comparison of the fit of the model to weekly influenza-positive SARI cases in all ages. Positive cases detected in the influenza surveillance system (black) with hypergeometric 95% confidence interval. Lines and shading represent the median (red) and 50% (green) and 75% credible intervals (blue) of the fitted model. Note that the model is fitted to age-specific data, but age groups are aggregated here for clarity. **a** Influenza B. **b** Influenza A(H3N2). **c** Influenza A(H1N1)pdm09). Influenza A(H1N1)pdm09) data from January 2010 to December 2012 were excluded from the analysis

We fitted 11 periods of high influenza activity in 7 of the 9 years of surveillance data. Five periods were associated with influenza B, four with A(H3N2) and two with A(H1N1)pdm09 (Fig. 2). In 1 year, there were 3 peaks in activity (September 2017–August 2018), and in 2 years, there were two peaks in activity (September 2010–August 2011, and September 2015–August 2016). September 2014–August 2015 and September 2016–August 2017 had no influenza activity periods that met the activity-period decision rule. The remaining 3 years had one period of influenza activity each (Table 4, Additional file 2 section 5).

**Table 4:** Periods of high influenza activity, 2010–2018

Year	NH season	Subtype	Vaccine match	SH season	Subtype	Vaccine match
Jan 2010–Aug 2010				03/2010–12/2010	A(H3N2)	M
2010–2011	12/2010–08/2011	B	M	08/2011–03/2012	B	M
2011–2012	12/2011–05/2012	A(H3N2)	U			
2012–2013				05/2013–12/2013	B	M
2013–2014	12/2013–09/2014	A(H1N1)pdm09	M			
2014–2015						
2015–2016	11/2015–05/2016	B	M	03/2016–11/2016	A(H3N2)	U
2016–2017						
2017–2018	09/2017–06/2018	B	U	06/2018–12/2018	A(H3N2)*	U
	01/2018–10/2018	A(H1N1)pdm09	M			

An influenza year begins in September and ends in August the following year. “M” means the vaccine was well matched to circulating strains (VE = 70%) [16]. “U” means vaccine was poorly matched to circulating strains (VE = 42%) [16]. Blank cells indicate no detectable peak in influenza activity. *There were no SH VE estimates available at the time, and we used VE values for the NH vaccine

Of the 11 periods of high influenza activity, 6 started between September and February and were suited to vaccination with the NH vaccine, and 5 started between March and August and were suited to the SH vaccine (Table 4). There were 8 instances where limited influenza activity did not meet the activity decision criteria (Additional file 2, section 5).

### Disease burden in the absence of vaccination

**Fig. 3:**
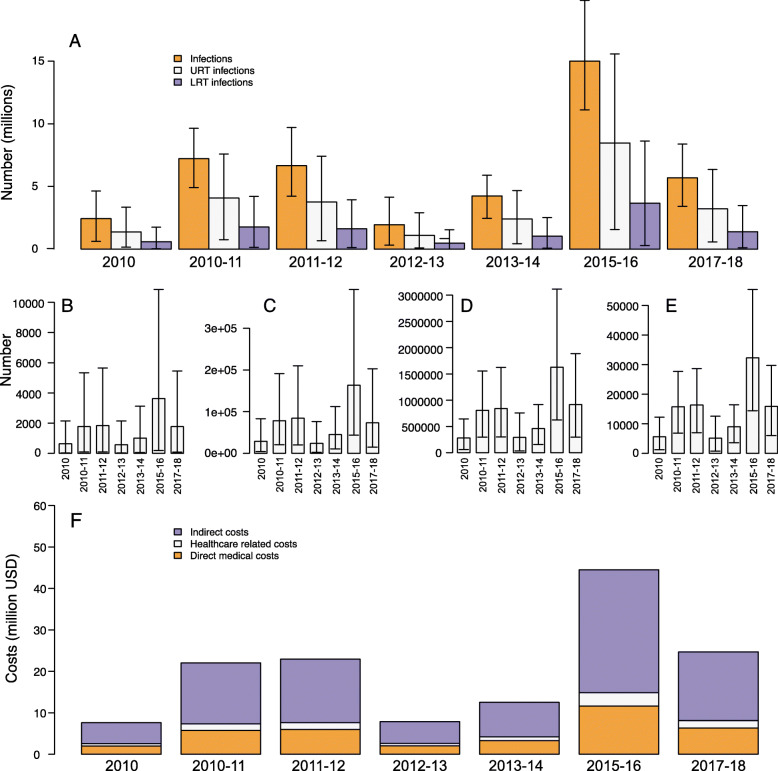
Influenza burden in the absence of vaccination in all age groups, 2010–2018. Mean and 95% credible interval shown for each calendar year (September–August). **a** Influenza infections, upper respiratory tract infections and lower respiratory tract infections. **b** Deaths. **c** DALYs. **d** Outpatient visits. **e** Hospitalisations. **f** Costs. Note that *y*-axes vary. There were three periods of high influenza activity in Sep 2017–Aug 2018, two periods of high influenza activity in Sep 2010–Aug 2011 and Sep 2015–Aug 2016. Years with no detectable periods of high influenza activity are not included in the figure

We estimated that the mean number of infections per year (includes asymptomatic and symptomatic infections) was 2.0–15.0 million (Fig. 3, and Additional file 3, table 1), corresponding to a mean annual attack rate of 5–32%. For years where more than one period of high influenza activity was modelled, the mean annual number of infections was 5.7–15.0 million and the mean annual attack rate was 12–32%, while for years that had one peak, the yearly average was 2.0–6.7 million infections with a mean annual attack rate of 5–16%.

We estimated that the average annual rate of infection was 4547–32,343 per 100,000 population. Rates of infection were highest among children 1–5 years (Additional file 3, table 2). There were 1.1–8.5 million upper respiratory tract infections, 0.5–3.7 million lower respiratory tract infections and 570–3626 deaths annually (Fig. 3). Deaths were highest in the 1–5 and >50 age groups. The average annual mortality rate was 1–8 per 100,000 population. The highest mortality rates were observed in the ≥50, <1 and 1–5 age groups (Additional file 3, table 2). There were 24,000–163,000 DALYs associated with influenza illness each year. Children 1–5 years of age consistently contributed the highest number of DALYs (Additional file 3, table 1).

There were 0.3–1.6 million outpatient visits and 5000–32,000 hospitalisations across all age groups each year (Fig. 3). The highest number of hospitalisations was observed among children 1–5 years of age (Additional file 3, table 3). The annual mean rate of hospitalisation across all ages was 12–70 per 100,000 population and was highest among children 1–5 years of age followed by those < 1 year of age (Additional file 3, table 2).

### Costs of influenza illness

We estimated that the direct medical costs associated with outpatient and inpatient care were $2.0–$11.6 million per year (Fig. 3 and Additional file 3, table 4). Of this amount, outpatient costs accounted for approximately three quarters of total direct medical costs. Healthcare-related costs amounted to $0.6–$3.2 million annually while indirect costs associated with lost wages and childcare costs equalled $5.1–$29.6 million per year. The mean annual total cost of influenza-associated illness was $20.3 million each year (annual average ranged between $7.6 and $44.5 million). Of note, indirect costs accounted for nearly 60% of all influenza-associated costs (Fig. 3 and Additional file 3, table 4).

### Comparison of vaccination strategies

**Fig. 4:**
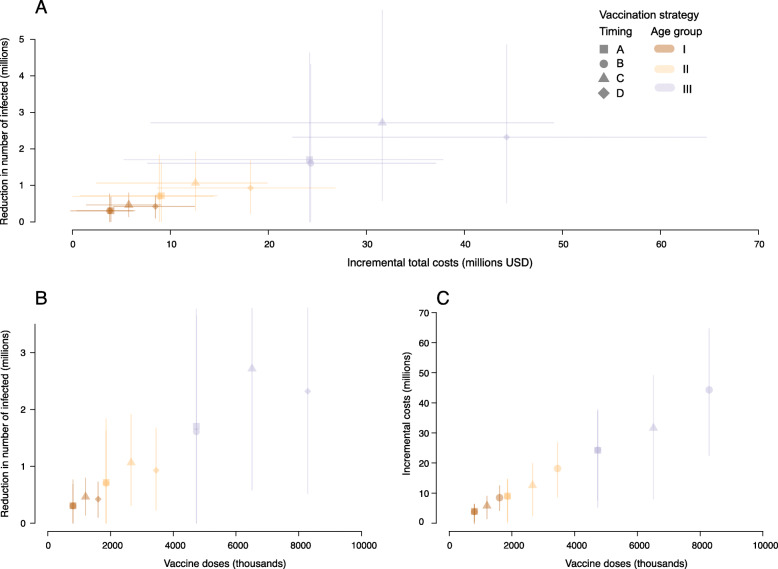
Summary of annual mean incremental cost, reductions in infections and vaccine doses per strategy. **a** Annual reduction in number of infections and incremental total societal costs per strategy. **b** Annual reduction in number of infections and vaccine doses per vaccine strategy. **c** Annual incremental total societal costs and vaccine doses costs per strategy. Strategies are vaccinating children 6–23 months (strategy I), 2–5 years (strategy II) and 6–14 years (strategy III) with either the Southern Hemisphere influenza vaccine (Strategy A) or Northern Hemisphere vaccine (Strategy B) or both (Strategy C: twice yearly 3-month vaccination periods, or Strategy D: year-round vaccination). The points mark posterior mean estimates and lines 95% credible intervals

There were substantial differences in mean costs and outcomes by strategy (Fig. 4). Mean annual vaccination purchase and administrative costs were lower when vaccinating children 6–23 months (strategy I: $4.3–$10.5 million) compared to 2–5-year-olds (strategy II: $9.9–$22.7 million) and 6–14-year-olds (strategy III: $25.3–$54.4 million) (Additional file 3, table 5). Total societal costs associated with vaccination and illness were lowest with strategy I as compared to the other strategies: it cost an annual average of $10.5–$53.4 million for strategy I, $14.5–$63.2 million for strategy II and $28.7–$87.8 for strategy III (Table 5). Strategy III (vaccinating 6–14 year olds) required the highest number of vaccine doses, resulted in the highest costs and led to the largest decrease in the number of infections (Fig. 4).

**Table 5:** Average annual total societal costs and 95% credible intervals (CIs) per vaccination strategy in millions of USD

	Strategy											
	I				II				III			
Year	A	B	C	D	A	B	C	D	A	B	C	D
2010	10.47 (4.99–22.4)	12.3 (5.58–26.8)	13.22 (7.12–26.02)	15.52 (9.08–28.28)	14.53 (9.47–23.82)	18.44 (10.87–33.1)	20.02 (13.42–31.01)	24.77 (17.12–36.28)	28.65 (21.06–38.28)	35.14 (24.28–51.56)	39.53 (29.07–52.79)	50.31 (37.05–66.96)
2010–11	25.7 (9.89–58.27)	25.32 (9.79–57.84)	27.2 (11.9–58.74)	29.96 (14.23–62.08)	30.6 (15.28–61.51)	29.75 (15.14–59.51)	33.38 (18.94–61.44)	39.04 (23.2–68.8)	44.11 (28.7–70.93)	44.27 (28.78–70.65)	50.86 (35.6–72.91)	63.32 (44.63–89.53)
2011–12	27.85 (10.44–63.18)	26.96 (10.19–61.17)	29.64 (12.57–64.32)	32.28 (14.84–67.11)	34.33 (16.4–69.85)	32.08 (15.76–64.48)	37.58 (20.31–70.94)	42.95 (24.54–77.14)	51.94 (31.52–88.39)	45.96 (29.32–74.06)	58.5 (38.79–89.58)	70.68 (48.1–104.96)
2012–13	10.96 (5.05–25.92)	12.94 (5.51–30.84)	13.93 (7.26–29.65)	16.59 (9.34–32.58)	15.3 (9.94–25.72)	19.59 (11.17–37.81)	21.21 (14.09–34.17)	26.68 (18.09–41.05)	30.86 (22.82–40.92)	37.68 (25.53–57.45)	42.45 (31.39–56.29)	54.35 (40.13–72.27)
2013–14	17.72 (7.9–37.94)	15.64 (7.3–32.6)	18.74 (9.82–36.49)	21.31 (12.07–39.21)	24.55 (14–44.98)	19.79 (12.29–33.19)	26.16 (17.02–41.7)	31.41 (21.16–47.5)	43.13 (29.18–64.84)	33.28 (24.47–44.45)	46.15 (33.91–61.7)	58.33 (43.01–77.66)
2015–16	48.3 (16.51–113.91)	48.47 (16.56–114.58)	50.31 (18.88–114.99)	53.37 (21.44–118.68)	52.91 (22.38–115.21)	53.61 (22.69–116.66)	56.94 (27.06–117.17)	63.21 (32.38–124.8)	64.18 (37.24–116.25)	68.06 (38.78–124.43)	73.27 (46.12–121.35)	87.82 (57.09–139.53)
2017–18	28.72 (10.86–68.26)	27.17 (10.44–64.33)	29.46 (12.91–65.93)	32.54 (15.56–69.14)	33.57 (17–68.92)	31.08 (16.29–61.99)	34.85 (20.51–63.09)	41.42 (25.5–71.71)	48.72 (32.39–75.87)	48.1 (31.97–74.92)	52.83 (38.48–70.92)	67.61 (49.24–90.92)

Strategies are vaccinating children 6–23 months (strategy I), 2–5 years (strategy II) and 6–14 years (strategy III) with either the Southern Hemisphere influenza vaccine (Strategy A) or Northern Hemisphere vaccine (Strategy B) or both (Strategy C: twice yearly 3-month vaccination periods, or Strategy D: year-round vaccination)

When vaccination was well timed/aligned to influenza activity, the annual mean ICER per DALY averted ranged between $749 and $1385 per DALY averted for strategy IA, $442 and $1877 for strategy IB, $678 and $4106 for strategy IC and $1147 and $7933 for strategy ID. For II strategies, it ranged between $945 and $1573 for strategy IIA, $563 and $1869 for strategy IIB, $662 and $4085 for strategy IIC and $1169 and $7897 for strategy IID. For III strategies, it ranged between $923 and $3116 for strategy IIIA, $1005 and $2223 for strategy IIIB, $883 and $4727 for strategy IIIC and $1467 and $6813 for strategy IIID (Additional file 3, table 6).

**Fig. 5:**
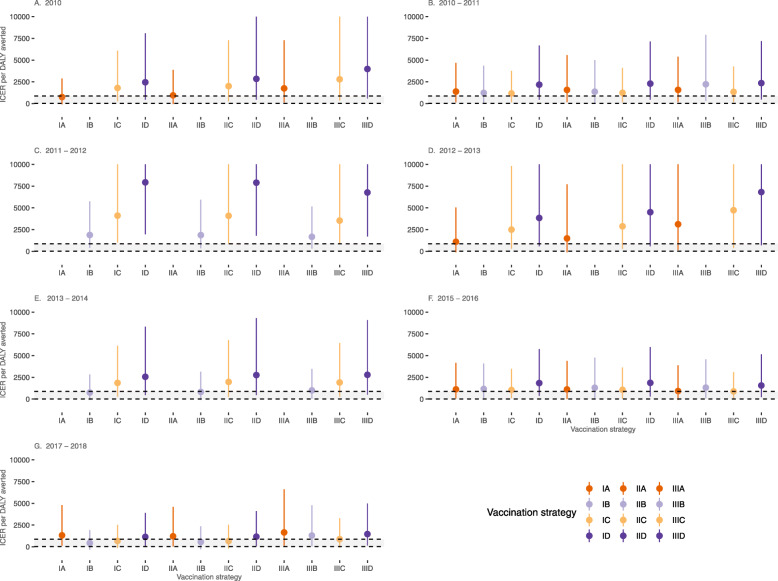
ICER per DALY averted and 95% CI. Results for 2014–15 and 2016–17 are not shown as there were no periods of high influenza activity detected in these years and calculation of ICER values per DALY averted would produce an infinite value as no DALYs would be averted. Similarly, ICER values are not shown for A and B strategies where vaccine administration was mistimed to influenza activity as vaccination was considered ineffective that year. Note the *y*-axes are cut off at 10,000 while actual values may exceed this value. Section shaded grey between the horizontal dotted lines represents outputs that fall within a willingness-to-pay threshold of 1–51% of the GDP per capita (i.e. between $17 and $872). Values below zero are cost saving. Strategies are vaccinating children 6–23 months (strategy I), 2–5 years (strategy II) and 6–14 years (strategy III) with either the Southern Hemisphere influenza vaccine (Strategy A) or Northern Hemisphere vaccine (Strategy B) or both (Strategy C: twice yearly 3-month vaccination periods, or Strategy D: year-round vaccination)

**Fig. 6:**
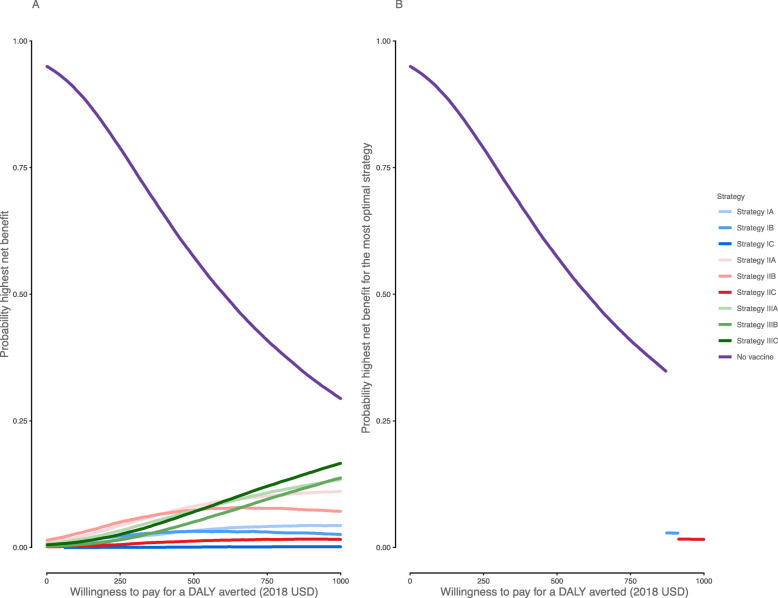
Cost-effectiveness acceptability curve and frontier for strategies with the highest incremental net monetary benefit. **a** Cost-effectiveness acceptability curve. **b** Cost-effectiveness acceptability frontier. NB: X axis is limited to 1000 USD per DALY averted. Strategies are vaccinating children 6–23 months (strategy I), 2–5 years (strategy II) and 6–14 years (strategy III) with either the SH influenza vaccine (Strategy A) or NH vaccine (Strategy B) or both (Strategy C: twice yearly 3-month vaccination periods, or Strategy D: year-round vaccination)

There was considerable overlap between ICER values obtained for each strategy in the 7 years with influenza activity (Fig. 5 and Additional file 3, table 6). Depending on the strategy, 0–3% of outputs were cost saving, and only 15–39% of outputs were equal to or less than the upper limit of the WTP threshold of $872 (51% of annual GDP per capita). Using the average INMB values, vaccination was not cost-effective in 2011–2012 at the tested WTP thresholds (Additional file 2, section 6). When comparing age groups, I strategies (vaccinating children 6–23 months of age) had the highest mean INMB values at the lowest WTP values in 5 of 7 years; however in 2015–2016, III strategies (vaccinating 6–14 year olds) had the highest mean INMB at the lowest WTP value (Additional file 2, section 6 and Additional file 4). In regard to timing of vaccination, A strategies (April–June) and B strategies (October–December) had the highest mean INMB at the lowest WTP values in an equal number of years. C strategies (vaccinating in two 3-month campaigns) were the most cost-effective strategies at the upper WTP limit ($872) in 2010–2011, 2015–2016 and 2017–2018; however, D strategies were never cost-effective at Kenya’s range of WTP values (Additional file 2, section 6). On average, across the 7 years of influenza activity, no vaccination strategy was cost-effective at $17 (lower limit of WTP range), and $445 (median value of WTP range). At $872 (upper limit of WTP range), strategy IB had the highest mean INMB; however, it had low probability (3%) of being the most optimal strategy (Fig. 6, Table 6).

**Table 6:** Incremental net monetary benefit values and probabilities for each vaccination strategy at a willingness-to-pay threshold of $872 per DALY averted

Strategy	Mean INMB value in ‘000 s	INMB 95% credible interval in ‘000 s		Probability of highest INMB benefit	Rank
		Lower quantile	Upper quantile		
Strategy IA	− 472	− 6201	10,054	4%	5
Strategy IB	3	− 5975	13,302	3%	1
Strategy IC	− 217	− 6976	13,545	0%	3
Strategy ID	− 3424	−10,351	9188	0%	8
Strategy IIA	− 1293	−14,377	23,996	11%	7
Strategy IIB	− 581	−13,854	28,726	7%	6
Strategy IIC	− 387	−15,597	31,947	2%	4
Strategy IID	− 7507	−22,440	20,834	0%	11
Strategy IIIA	− 7077	−36,674	53,262	12%	10
Strategy IIIB	− 7531	−35,320	44,647	12%	12
Strategy IIIC	− 3633	−39,244	70,255	14%	9
Strategy IIID	− 20,326	−54,467	42,876	0%	13
No vaccine	0	–	–	35%	2

*INMB* incremental net monetary benefit. Strategies are vaccinating children 6–23 months (strategy I), 2–5 years (strategy II) and 6–14 years (strategy III) with either the Southern Hemisphere influenza vaccine (Strategy A) or Northern Hemisphere vaccine (Strategy B) or both (Strategy C: twice yearly 3-month vaccination periods, or Strategy D: year-round vaccination)

The most favourable vaccination strategy each year was the same regardless of whether total societal costs or only direct medical costs were considered (Additional file 5); however, the WTP value at which vaccination became cost-effective was higher with direct medical costs (Additional file 2, section 6). Based on the average INMB values across the 7 years of influenza activity, no vaccination strategy was cost-effective at the upper limit of the WTP threshold when only direct medical costs were considered.

### Sensitivity analysis

Removing time discounting led to a 49–50% reduction in mean ICER per DALY averted across all strategies, and addition of social weighting led to a slight decrease (5–6%) in mean ICER value. At a vaccine purchase price of $1.50 USD, the mean ICER decreased by 44–62%. At a vaccine purchase price of $10.0 USD, the mean ICER value increased by 144–178%, and at $6.0 USD, the mean ICER value increased by 38–43%, while at a vaccine purchase price of $4.50 USD the mean ICER value increased by 31–38%. Maintaining a uniform vaccine coverage across all age groups led to a 1–4% decrease in mean ICER value for strategies targeting children 2–5 years, and a 7–20% reduction in mean ICER values for strategies targeting children 6–14 years. Using the INMB approach, no vaccination strategy was cost-effective at a vaccine price of $4.5 USD and above. For all other sensitivity analysis outputs, vaccinating children 6–23 months of age with the NH vaccine (strategy IB) remained the most cost-effective strategy.

## Discussion

There were yearly variations in peaks of influenza activity, with at most three periods of increased influenza activity each year. Rates of infection and hospitalisation were highest in the 1–5 year age group, while mortality rates were highest in individuals ≥50 and children <5. As a result, children 1–5 years contributed the highest number of DALYs each year. Given the expected vaccine coverage levels, we found that targeting children 6–23 months in an annual 3-month campaign was the most favourable of the vaccination strategies, although the probability of any vaccination strategy being cost-effective, even at the upper limit of the WTP threshold, was low. Vaccination was most cost-effective when vaccine was well matched to circulating strains and influenza activity occurred after hypothetical vaccination campaigns. Vaccinating children 6–23 months of age was least expensive although the reduction in number of infections was not as substantial as those observed at higher coverage levels attainable by vaccinating more children in older age groups. The provisional KENITAG recommendation to vaccinate children 6–23 months would be the least expensive strategy for the government to adopt and frequently had the highest INMB, although at very low probabilities.

Primary school-going children have the highest contact rates in Kenya [22], and therefore, vaccinating this group could yield considerable benefit in all ages due to indirect protection [36]. We found that the overall reduction when vaccinating children 6–14 years old was only more favourable than vaccinating children 6–23 months of age (who have a higher burden of severe disease) in 2015–2016 when the vaccine was poorly matched to circulating strains. Vaccinating school-going children may be an important strategy when inadequate protection levels are attained in those most susceptible to severe disease [37]. Nonetheless, we found that when the vaccine is well matched to circulating strains, direct protection of the age group with highest burden of severe disease was most favourable. Studies have previously shown that with lower vaccine efficacy the indirect benefit of vaccination exceeds the direct benefits by larger margins [13] and could explain why we obtained more favourable values when vaccinating children 6–14 years of age in years when vaccine effectiveness was lower.

We found that year-round vaccination was always the least cost-effective strategy. For this strategy, vaccination after infection was more likely to occur. Therefore, vaccinating in short 3-month campaigns was a more favourable strategy than year-round vaccination. These findings highlight the need to vaccinate as much of the target population as possible as soon as the influenza vaccine becomes available in order to enjoy the full benefit of vaccination in Kenya. However, evidence of intraseasonal waning immunity and its implications on vaccination timing may lead to changes in recommendations for vaccination timings in future [38–40].

Vaccinating twice a year was most cost-effective at higher WTP values. This strategy would ensure that a proportion of the population has some protection against the currently circulating influenza strains by using the most up-to-date vaccine formulation. Continuous mutations in nucleic acids coding for influenza antigens lead to semi-annual reviews of the components used for the production of influenza vaccine [41]. Over the past 10 years, the strains in the NH vaccine differed from the incoming SH vaccine in 6 years and differed from the contents of the preceding SH vaccine in 3 years [42]. Despite the possibility of waning protection [43–45], vaccinating once per year should provide some protection over a 12-month period if there is no need for a change in the composition of the NH and SH vaccine. Once-a-year vaccination in short 3-month campaigns should be considered; however, surveillance is needed to monitor whether the vaccine is well matched to circulating strains that circulate in the latter half of the year.

In this study, most of the model outputs were unlikely to be cost-effective given a willingness-to-pay threshold of 1–51% of the GDP. These findings were contingent on a vaccine price per dose of $3, which is lower in price than vaccine available in high-income countries [46]. Very few outputs (1% of all simulations) at the price of $3 were cost saving. Overall, vaccination was unlikely to be cost-effective; however, if we had used previous WHO thresholds for cost-effectiveness of health interventions (i.e. (i) very cost-effective if less than the annual GDP per capita, (ii) cost-effective if 1–3 times the GDP, and (iii) not cost-effective if greater than 3 times the GDP [47]), we would have found that vaccination was most likely very cost-effective or cost-effective regardless of the strategy modelled. However, there is debate over the suitability and affordability of the WHO threshold for cost-effectiveness of interventions in LMICs [47, 48] and as an alternative a threshold of 1–51% GDP per capita for LMICs has been proposed [34]. This lower threshold is postulated to better reflect the constraints within the “supply side” of healthcare funding and considers the opportunity costs of the choice of interventions [34]. Selecting an appropriate ICER threshold value is critical: if interventions that cost more than the appropriate ICER threshold are implemented, they result in a net reduction in health, as more health benefits could be gained by choosing interventions of a lower ICER value [49].

Influenza vaccination of children 6–23 months age was cost-effective at a WTP value of $872 per DALY averted, while vaccines already included in the Kenya expanded programme on immunisation (EPI) have considerably lower ICER values. For example, rotavirus vaccine and *Haemophilus influenzae* type b vaccine cost approximately $38 per DALY averted [50, 51], while the pneumococcal vaccine costs $59 per DALY averted [52]. Continuing the pneumococcal vaccination programme beyond 2022–2027 when Kenya transitions to the full Gavi price ($3.05 per dose) would still result in a cost per DALY averted of $153 (95% prediction intervals of $70–$411) [53].

In 2010, half of the vaccines provided or considered for provision in LMICs cost less than $100 per DALY averted, 25% cost between $100 and $500 per DALY averted and 9% cost between $500 and $1000 per DALY averted (2010 dollar values) [54]. A decrease in vaccine price and improved influenza vaccine effectiveness, duration of protection, and if possible, long-term immunity would increase influenza vaccine cost-effectiveness and likely adoption in LMICs [55].

Although the range of ICER values per DALY averted were similar among the strategies, the modelled vaccination strategies substantially differed in vaccine purchase costs. Over the past 3 years (2016–2019), the government of Kenya allocated approximately $7 million per financial year to the immunisation programme, while Gavi contributed $26 million per year [56]. We estimated that at a vaccine price of $3 per dose, the government would spend $4.3–$10.5 million in vaccine purchase costs for the least expensive strategy, and $25.3–$54.4 million for the most expensive strategy, which represents a significant proportion of the immunisation budget. Cost differences between strategies could therefore influence selection of vaccination strategy.

Our findings need to be interpreted in light of several limitations. In 2014–2015 and 2016–2017, influenza activity did not meet our decision rule for periods of activity. These periods were characterised by nationwide healthcare worker strikes in public hospitals and disruptions in influenza surveillance funding (2014–2015), both of which plausibly led to a decrease in the number of influenza-positive SARI patients detected. Therefore, our model could have underestimated the burden of influenza and the impact and cost-effectiveness of seasonal influenza vaccination. Indeed, we were not able to fit all the observed periods of influenza activity, which could also underestimate the impact of vaccination over those periods. More complete and robust surveillance data could improve estimates and give further confidence in the findings presented here.

We limited our burden calculation to periods of high influenza activity. However, the overall rates of hospitalisation across all ages are comparable to past estimates of national disease burden conducted in Kenya covering similar years [1, 57]. Using a simpler methodology that took into account influenza activity throughout the year, we previously estimated the mean annual rates of influenza-associated hospitalised SARI to be 21 (95% confidence limit 19–23) per 100,000 population over the period January 2012–December 2014 [1]. For a similar period in our study, September 2011–August 2014, we estimated the mean annual influenza hospitalisation rates to be 24 per 100,000. These similar results may be explained by the fact that the identified periods of high influenza activity frequently lasted 6 or more months, and likely captured most of the annual influenza activity. Although we focussed on peaks in activity, we obtained comparable rates of illness to studies that considered year-round activity. However, our rates of hospitalised influenza during the early years of this study were slightly lower than previous estimates from August 2010 to July 2011, when published rates of influenza-associated hospitalised SARI were 70 (95% confidence limits 50–90) per 100,000 persons [57]. Our estimated rate for the period September 2010–August 2011 was 39 (95% credible interval 17–69) per 100,000 persons. This disparity may be explained by the fact that we excluded circulation of A(H1N1)pdm09 from January 2010 to December 2011 during our analysis. We excluded the first 2 years of circulation of the pandemic strain because these epidemics were not a typical influenza season—there was no single peak and numerous small epidemics occurred. This exclusion could have led to underestimation of vaccine cost-effectiveness if vaccines were matched to the pandemic strain during this time. Additionally, if competitive viral interaction were occurring, the circulation of A(H1N1)pdm09 could have suppressed A(H3N2) or B epidemics, leading to underestimation of vaccine impact.

In this analysis, we assumed that vaccination with SH or NH vaccine did not protect against transmission starting in the alternate hemisphere’s vaccination period. This assumption was informed by the potential for waning immunity suggested by declining vaccine antibody titres [58, 59] and/or possible mismatch of vaccine composition to circulating subtypes [42]. If there were lasting protection [60], there would be a higher impact of the vaccine in later seasons and an increase in cost-effectiveness. On the other hand, for periods of influenza activity that fell within a particular vaccination period, we also assumed protection was maintained at a constant level for the duration of influenza activity, regardless of whether the epidemic ran into the next vaccination period, i.e. even when periods of influenza activity lasted more than 6 months. This could lead to overestimation of the impact of vaccination.

We adopted a societal perspective for costs. However, we did not have local data on over-the-counter medication costs, lost wages and childcare costs for non-medically attended symptomatic influenza cases, and these costs were not incorporated in the analysis. The lack of local data may have led to underestimation of costs as well as the benefits of vaccination. We assumed vaccine completely protects a proportion of those vaccinated, i.e. “all-or-nothing” protection. However, where the influenza vaccine does not prevent infection, it may still reduce the severity and duration of illness [61] which could have underestimated the benefit of vaccination. Finally, we did not include non-respiratory influenza illness in our analysis. Although they are important manifestations of severe influenza, they contribute only 7% of the total costs of illness and may not have made significant differences to the ICER values [62].

## Conclusion

Influenza vaccination of children 6–23 months of age once per year was the most favourable vaccination strategy; however, it is unlikely to be a cost-effective intervention using a WTP threshold of 1–51% of annual GDP per capita. Targeting children in older age groups led to the largest reduction in the number of cases but was not necessarily the most cost-effective strategy at our WTP threshold. Further reductions in cost per dose and improvements in vaccine effectiveness and long-term immunity would make the influenza vaccine more attractive for inclusion in the EPI.

## Supplementary information

**Additional file 1.** Influenza surveillance data 2010–2018.

**Additional file 2.** Supplementary text.

**Additional file 3.** Additional tables showing: average number of influenza associated disease states and DALYs with their 95% credible interval limits, (2010–2018) (table on sheet 1); average annual rate per 100,000 of influenza associated disease states with their 95% credible interval limits, (2010–2018) (table on sheet 2); average number of influenza associated health care utilisation events with their 95% credible interval limits, (2010–2018) (table on sheet 3); national cost of influenza associated illness across all ages, (2010–2018) (table on sheet 4); average annual costs and 95% credible intervals (CIs) per vaccination strategy in millions of USD (table on sheet 5); incremental cost-effectiveness ratio for influenza vaccination using total societal costs (table on sheet 6).

**Additional file 4.** Yearly cost-effectiveness acceptability curves and frontiers for strategies with the highest incremental net monetary benefit considering total societal costs. NB: X axis is limited to 1000 USD per DALY averted. Strategies are vaccinating children 6–23 months (strategy I), 2–5 years (strategy II) and 6–14 years (strategy III) with either the SH influenza vaccine (Strategy A) or NH vaccine (Strategy B) or both (Strategy C: twice yearly 3-month vaccination periods, or Strategy D: year-round vaccination).

**Additional file 5.** Yearly cost-effectiveness acceptability curves and frontiers for strategies with the highest incremental net monetary benefit considering direct medical costs only. NB: X axis is limited to 1000 USD per DALY averted. Strategies are vaccinating children 6–23 months (strategy I), 2–5 years (strategy II) and 6–14 years (strategy III) with either the SH influenza vaccine (Strategy A) or NH vaccine (Strategy B) or both (Strategy C: twice yearly 3-month vaccination periods, or Strategy D: year-round vaccination).

## Data Availability

All data generated or analysed during this study are included in this published article and its supplementary information files.

## Abbreviations

A(H1N1)pdm09: Influenza A H1N1pdm09
A(H3N2): Influenza A H3N2
CDC: Centers for Disease Control and Prevention
CI: Credible intervals
DALY: Disability-adjusted life year
EPI: Expanded programme on immunisation
ICER: Incremental cost-effectiveness ratio
INMB: Incremental net monetary benefit
KENITAG: Kenya National Immunisation Technical Advisory Group
LMICs: Low- and middle-income countries
LRT: Lower respiratory tract
MCMC: Markov chain Monte Carlo
NH: Northern Hemisphere
NP: Nasopharyngeal
OP: Oropharyngeal
rRT-PCR: Real-time reverse transcription-polymerase chain reaction
SARI: Severe acute respiratory illness
SEIR: Susceptible-Exposed-Infectious-Recovered
SH: Southern Hemisphere
URT: Upper respiratory tract
VE: Vaccine effectiveness
WHO: World Health Organization
WTP: Willingness-to-pay
