# Supplementary material for: Seasonal influenza vaccination in Kenya: an economic evaluation using dynamic transmission modelling
Source: BMC Med. 2020 Aug 20;18:223. doi: 10.1186/s12916-020-01687-7 (PMC7438179; doi:10.1186/s12916-020-01687-7)
Supplement: Supplementary file 2 — Additional file 2. Supplementary text. [file 12916_2020_1687_MOESM2_ESM.pdf]

Supplementary text for

Seasonal influenza vaccination in Kenya: an economic evaluation using dynamic transmission modelling

Jeanette Dawa*[1,2], Gideon O. Emukule[3], Edwine Barasa[4], Marc Alain Widdowson[5,6], Omu Anzala[1], Edwin Van Leeuwen[7], Marc Baguelin[8,9], Sandra S. Chaves[3,10], Rosalind M. Eggo[8]

1. KAVI-Institute of Clinical Research, College of Health Sciences, University of Nairobi, Nairobi, Kenya
2. Washington State University Global Health Programs Kenya Office, Nairobi, Kenya
3. Influenza Program, Centers for Disease Control and Prevention, Nairobi, Kenya
4. Health Economics Research Unit, KEMRI Wellcome Trust Research Programme, Nairobi, Kenya
5. Division of Global Health Protection, Center for Global Health, Centers for Disease Control and Prevention, Nairobi, Kenya
6. Division of Global Health Protection, Center for Global Health, Centers for Disease Control and Prevention, Atlanta, United States
7. Public Health England, London, United Kingdom
8. London School of Hygiene & Tropical Medicine, London, United Kingdom
9. Imperial College London, United Kingdom
10. Influenza Division, National Center for Immunization and Respiratory Diseases, US Centers for Disease Control and Prevention, Atlanta, Georgia, United States

Table of Contents

1 Further information on health facilities within influenza surveillance system 3
1.1 Map of influenza surveillance system 3
1.2 Defining the catchment population 3
1.3 Summary of surveillance data from health facilities 4
2 Further details on the epidemiological model 5
2.1 Age and risk groupings in modelling framework 7
2.2 Data inputs 8
3 Vaccine effectiveness values for each vaccination period for modelled influenza seasons 11
4 Further information on the economic evaluation 12
4.1 Economic evaluation decision tree 12
4.2 Additional inputs in the economic model 13
5 Further information on fitted model 14
5.1 Fitted periods of influenza activity 14
5.2 Periods that did not meet decision rule criteria 15
5.3 Fit of model to data and distribution of posteriors 16
5.3.1 Influenza B 16
5.3.2 Influenza A(H3N2) 21
5.3.3 Influenza A(H1N1)pdm09 25
6 Additional results 27
6.1 Results of incremental net monetary benefit analysis 27
6.2 Results of sensitivity analysis 28
6.2.1 Social weighting and time discounting 28
6.2.2 Changes in vaccine price 29
6.2.3 Changes in vaccine coverage 30
7 Comparison between UK model and Kenya model 31
8 References 32

1 Further information on health facilities within influenza surveillance system

1.1 Map of influenza surveillance system

From 2010 to 2018 the Centers for Disease Control and Prevention (CDC) in collaboration with the Ministry of Health conducted influenza surveillance in Kenya at each of the largest public health facilities in the following counties: Kakamega, Siaya, Nyeri, Nakuru, Mombasa, and Nairobi. Influenza surveillance also took place within refugee camps. The analysis was limited to data from the county referral hospitals (CRH) in Kakamega, Siaya, Nyeri, Nakuru and Mombasa from where we were able to define catchment populations.

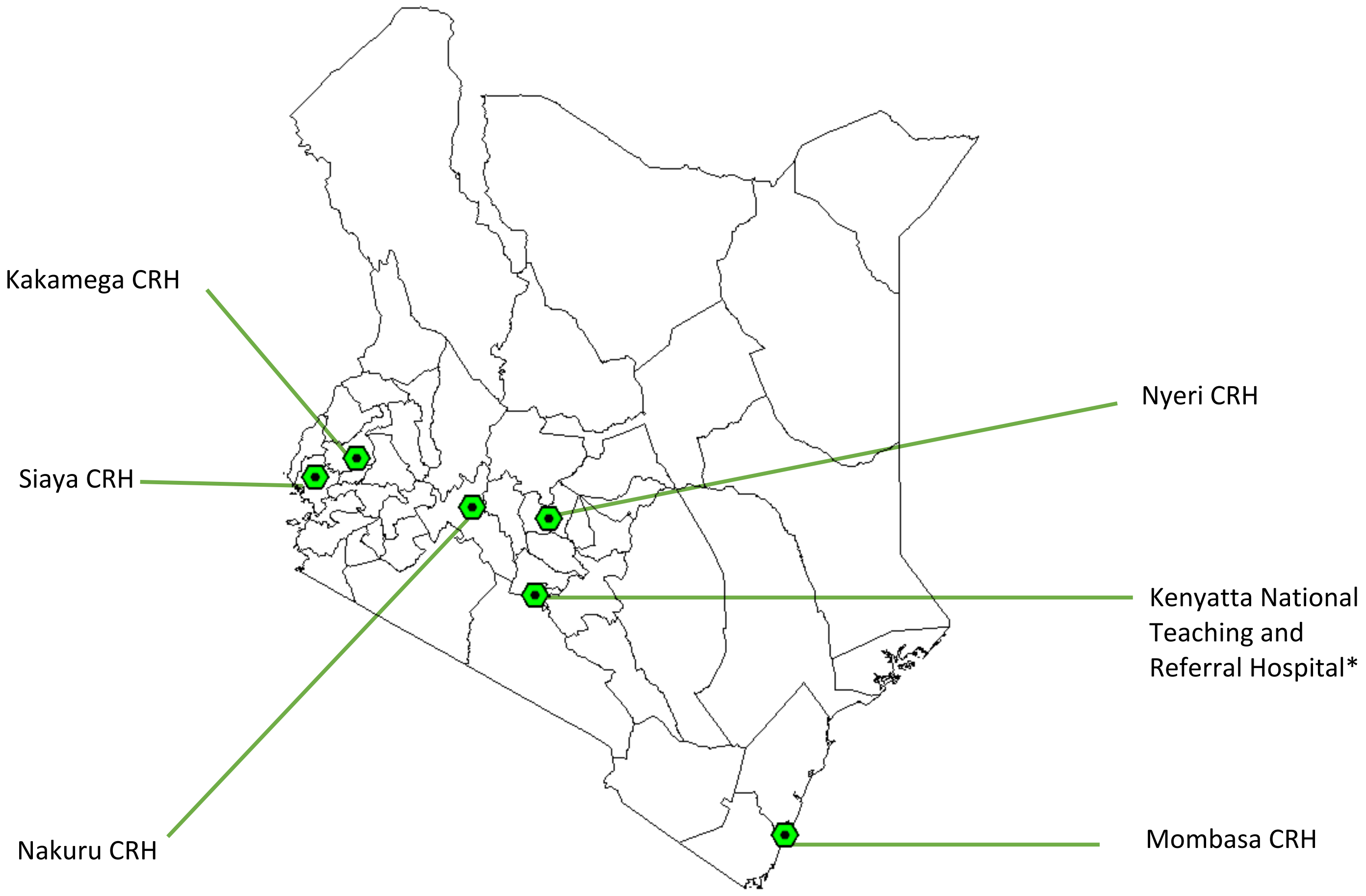

*Additional file 2 figure 1: Map of influenza surveillance sites in Kenya. CRH – county referral hospital. *Data from Kenyatta National Teaching and Referral Hospital not included in the analysis.*

1.2 Defining the catchment population

We obtained Kenyan age group specific population density data for the years 2010 and 2015 (1). We plotted each sentinel site on ArcGIS (ESRI) using its longitude and latitude (Additional file 2 table 1) and calculated the catchment population within a 10 kilometre radius of each health facility. This was

informed by a local study that showed that ninety percent of children admitted in a health facility with symptoms of a febrile illness, reside within 10 kilometres of the health facility (2).

The annual population for each age group was estimated by assuming a constant growth rate between 2010 and 2015. The 2016 to 2018 population was obtained by applying the World Bank annual population growth estimate (3). We then assumed that only 5-20% of ill patients within the whole catchment population requiring hospitalisation were admitted at the county referral hospital, given the low levels of health care seeking (4) and presence of alternative inpatient health facilities within the community. Although Kenyatta National Teaching and Referral Hospital is part of the influenza surveillance system, using a 10-kilometre radius round this facility to define its catchment population is not appropriate. The national hospital serves a much larger population than the other county referral hospitals in the influenza surveillance system. For this reason, data from Kenyatta National Teaching and Referral Hospital was excluded from the model.

1.3 Summary of surveillance data from health facilities

For the period 2010 to 2018 there were 24,480 cases of severe acute respiratory illness (SARI) identified across the five surveillance sites. Of these cases, 80% (19,547) had respiratory samples tested for the presence of influenza. The influenza virus was detected in 8.6% (1,690) of samples tested.

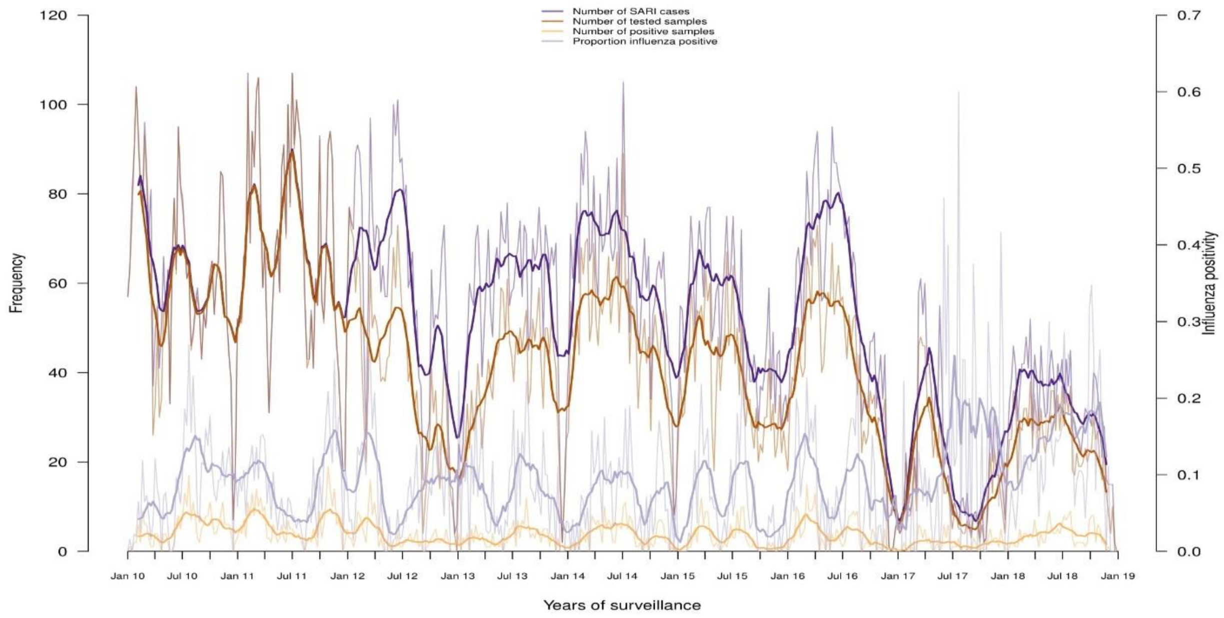

Additional file 2 figure 2: Weekly number of severe acute respiratory illness (SARI) cases, tested samples, positive samples (on left axis) and proportion of tested samples that were influenza positive (right axis) that were identified across the surveillance sites over the period 2010 to 2018. Darker lines represent the rolling mean. Lighter lines represent the weekly number/proportion.

2 Further details on the epidemiological model

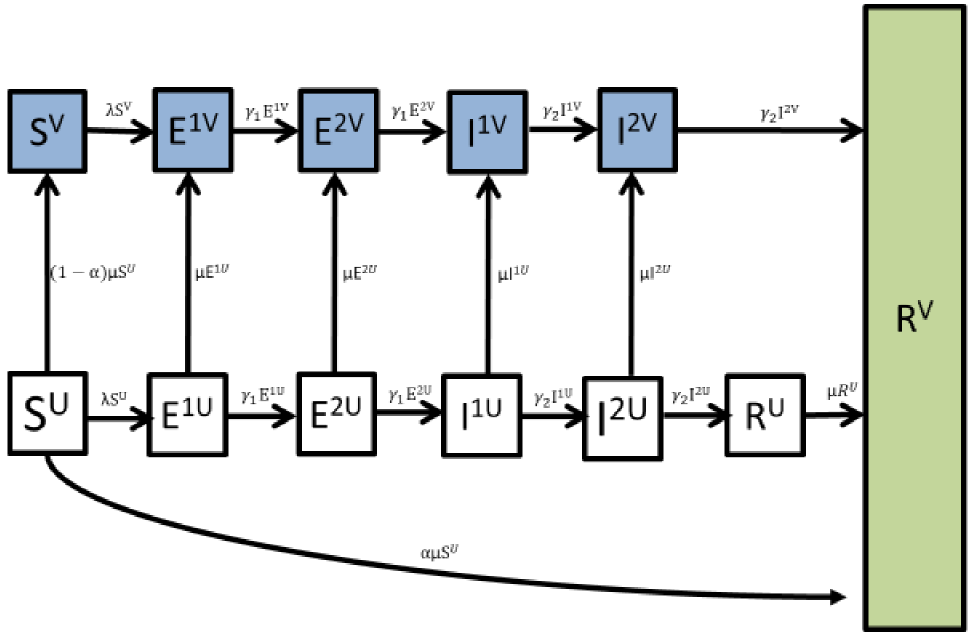

Additional file 2 figure 3: Epidemiological model of influenza transmission (5). S = susceptible population; E^1= first compartment of the exposed population; E^2= second compartment of the exposed population; I^1 = first compartment of infectious population; I^2 = second compartment of the infectious population; R = recovered population; V = vaccinated population; U =unvaccinated population; α = vaccine effectiveness; λ =the force of infection; γ_1 = rate of onset of infectiousness; γ_2 = recovery rate, μ = vaccination rate

The model uses a basic Susceptible-Exposed-Infectious-Recovered (SEIR) structure with two E and I compartments (SEEIIR structure). This was adopted to make the latent and infectious periods gamma distributed, rather than exponential (5). The differential equations of the transmission model are provided below while full details of the model are provided in Baguelin, 2013 and van Leeuwen, 2017 (5, 6):

$$\frac{dS_{ik}^{U}}{dt} = -\lambda_i S_{ik}^{U} - \mu_{ik} S_{ik}^{U} \quad ; \quad \frac{dS_{ik}^{V}}{dt} = -\lambda_i S_{ik}^{V} + (1-\alpha_i)\mu_{ik} S_{ik}^{U}$$

$$\frac{dE_{ik}^{1U}}{dt} = \lambda_i S_{ik}^{U} - \gamma_1 E_{ik}^{1U} - \mu_{ik} E_{ik}^{1U} \quad ; \quad \frac{dE_{ik}^{1NU}}{dt} = \lambda_i S_{ik}^{V} - \gamma_1 E_{ik}^{1V} + \mu_{ik} E_{ik}^{1U}$$

$$\frac{dE_{ik}^{2U}}{dt} = \gamma_1 (E_{ik}^{1U} - E_{ik}^{2U}) - \mu_{ik} E_{ik}^{2U} \quad ; \quad \frac{dE_{ik}^{2V}}{dt} = \gamma_1 (E_{ik}^{1V} - E_{ik}^{2V}) + \mu_{ik} E_{ik}^{2U}$$

$$\frac{dI_{ik}^{1U}}{dt} = \gamma_1 E_{ik}^{2U} - \gamma_2 I_{ik}^{1U} - \mu_{ik} I_{ik}^{1U} \quad ; \quad \frac{dI_{ik}^{1V}}{dt} = \gamma_1 E_{ik}^{2V} - \gamma_2 I_{ik}^{1V} + \mu_{ik} I_{ik}^{1U}$$

$$\frac{dI_{ik}^{2U}}{dt} = \gamma_2 (I_{ik}^{1U} - I_{ik}^{2U}) - \mu_{ik} I_{ik}^{2U} \quad ; \quad \frac{dI_{ik}^{2V}}{dt} = \gamma_2 (I_{ik}^{1V} - I_{ik}^{2V}) + \mu_{ik} I_{ik}^{2U}$$

$$\frac{dR_{ik}^{U}}{dt} = \gamma_2 I_{ik}^{2U} - \mu_{ik} R_{ik}^{1U} \quad ; \quad \frac{dR_{ik}^{1V}}{dt} = \gamma_2 I_{ik}^{2V} + \mu_{ik} (R_{ik}^{U} + \alpha_i S_{ik}^{U})$$

(1)

Where,

S	=	susceptible population
E^1	=	first compartment of the exposed population
E^2	=	second compartment of the exposed population
I^1	=	first compartment of infectious population
I^2	=	second compartment of the infectious population
R	=	recovered population
V	=	vaccinated population
U	=	unvaccinated population
i	=	age class
k	=	risk group
λ	=	the force of infection
γ_1	=	rate of onset of infectiousness
γ_2	=	recovery rate
μ	=	vaccination rate
α	=	vaccine effectiveness

2.1 Age and risk groupings in modelling framework

Additional file 2 table 1: Age and risk groups in model framework

Data	Values	Basis
Age groups	<1 year<br>1-5 years<br>6-14 years<br>5-19 years<br>20-49 years<br>≥ 50 years	Based on age groupings used in Kenyan contact survey (7)
Age specific susceptibility profiles	<15 years<br>15-49 years<br>≥50 years	Three age groups used to avoid overfitting of data. The age groups were identified over the process of fitting the model to the data.
Ascertainment probability age groupings	<1 year<br>1-5 years<br>≥6 years	Three age groups used to avoid overfitting of data. The age groups were identified over the process of fitting the model to the data. These age groupings had least correlation between parameters. Due to difference in healthcare seeking behaviour between age groups, the best fit of model to the observed data was obtained by allowing children <1 and 1-5 years to have their own ascertainment probability values, rather than maintaining the same susceptibility age groupings.

The fluEvidenceSynthesis package allows specification of high-risk groups within age groups. Unfortunately, there were limited national data on the proportions of each age group that were high risk, and thus all individuals were considered equally at risk of severe outcomes

2.2 Data inputs

Additional file 2 table 2: Assumptions of main data inputs in adapted Baguelin framework

Data	Source	Assumptions
Weekly SARI counts stratified by age group	KEMRI/CDC influenza surveillance data, 2010-2018 from Siaya, Kakamega, Nakuru, Nyeri and Mombasa influenza sentinel surveillance sites	Data adequately represents influenza activity in the country. Data from Kenyatta National Teaching and Referral Hospital was not included because the catchment population of the national referral hospital could not be adequately estimated.
Weekly virological data stratified by age group	KEMRI/CDC influenza surveillance data, 2010-2018 from Siaya, Kakamega, Nakuru, Nyeri and Mombasa influenza sentinel surveillance sites	As above
Population size by age	2009 population census data projected to the years under study using the world bank annual population growth rates	Population growth is uniform across regions and age groups
Contact data by age group	(7)	Contact patterns of rural and semi-urban Kilifi, Kenya are similar to the rest of Kenya. Contact patterns are constant throughout the year and do not vary between dry and wet seasons or school terms and school holidays
Monitored population around each influenza sentinel surveillance site	World population map density data projected to the years under study using world bank annual population growth rates	Population within a 10 kilometre radius of each health facility represents the catchment population of the health facility. It is informed by Noor, 2003 that states 90% of admissions within a health facility arise from the population within 10 km of the health facility (2)
Weekly monitored population	World population map density data projected to the years under study using world bank annual population growth rates	A random value with a minimum value from the expected population at the start of the season and a maximum value from the expected population at the end of the season given a uniform increase in population size throughout the year
Vaccine effectiveness	Published literature	Among those who are effectively vaccinated, protection is assumed to be complete whereas those who are not effectively vaccinated carry the same risk of infection as non-vaccinated individuals

Additional file 2 table 3: Data sources and assumptions for priors in epidemiological model

Data	Value	Source	Notes
**1. Ascertainment probability priors**			
<1 year of age	Log normal distribution; mean log -4.856275807, SD log 0.85064645	Refer to Additional file 2 table 5	The priors on ascertainment probability are generated by combining the mean and ranges of the 5 constituent probabilities, i to v given in Additional file 2 table 4.
1-5 years of age	Log normal distribution; mean log -4.913483683, SD log 0.85956516		
≥6 years of age	Log normal distribution; mean log -5.319344699, SD log 0.981261173		
**2. Susceptibility prior**			
0-14 years age group	Normal distribution, mean = 0.6, SD = 0.1	Assumption	
15-49 and ≥50 years age groups	No prior provided		
**3. Transmissibility prior**			
All ages	Normal distribution, mean = 0.165, SD = 0.055	Assumption	Uses the UK values on transmissibility Baguelin, 2013 (8) but incorporates a SD that is twice as wide

SD = standard deviation

Additional file 2 table 4: Data sources and assumptions for ascertainment probability priors in epidemiological model

Component of ascertainment probability prior	Mean and 95% confidence limit	Source	Notes
i. Probability of an infected case developing lower respiratory tract (LRT) symptoms	0.21(0.14-0.303)	(8)	We assume that the probability of severe infection is the same across influenza strains and sub-types
ii. Probability of a case with LRT being hospitalised			
<1 year of age	0.26(0.159-0.396)	(4)	
1-5 years of age	0.24(0.14-0.37)	Calculated	Based on the data from (4)
≥6 years of age	0.16(0.08-0.29)	(4)	
iii. Probability of people within a 10 km radius of the surveillance site being hospitalised at the surveillance site	0.125(0.05-0.2)	Assumption	
iv. Probability of being picked up by surveillance officer			
0-5 years of age	0.7(0.6-0.8)	Assumption	

≥6 years of age	0.5(0.3-0.7)	Assumption	The assumption is based on the fact that in the surveillance sites, the surveillance officers aim to record every case of SARI, however it is likely that during weekends/staff changes/staff absence a few cases may be missed. The robustness of surveillance is assumed to be 0.7(0.6-0.8) in children ≤5 years of age and 0.5(0.3-0.7) in older individuals. NB: We later take into account that not all SARI cases are tested when we fit the modelled data to the 'observed data' where in this case 'observed data' is the number of positive cases we would expect to see if all cases were tested.
v. Probability of a positive influenza case testing positive	0.55(0.3-0.8)	Assumption	The assumption is informed by the Feikin 2013 (9) paper that showed that approximately 48-74% of samples that were positive for influenza by either PCR or serology were positive by PCR.

3 Vaccine effectiveness values for each vaccination period for modelled influenza seasons

Northern hemisphere and southern hemisphere vaccine effectiveness (VE) was assumed to be either good (70% VE) or poor (42% VE) in all target age groups based on published estimates of vaccine effectiveness. If VE was ≥50% the vaccine was considered well matched to the circulating strain and unmatched if vaccine effectiveness was <50%.

Additional file 2 table 5: Vaccine effectiveness values for each vaccination period for modelled influenza season

Year	Subtype	Vaccine effectiveness (95% confidence interval)	Matched (M) or Unmatched (U)	Source
**Northern Hemisphere vaccine match to circulating strains**				
2010/2011	B	50% (14-71%)	M	(10)
2011/2012	A(H3N2)	39% (23-52%)	U	(11)
2013/2014	A(H1N1)pdm09	54% (46-61%)	M	(12)
2015/2016	B	55% (44-64%)	M	(13)
2017/2018	A(H1N1)pdm09	67% (54-76%)	M	(14)
2017/2018	B	42% (25-56%)	U	(14)
**Southern hemisphere vaccine match to circulating strains**				
2010	A(H3N2)	72% (−26–94%)*	M	(15)
2011	B	72% (−26–94%)*	M	(15)
2013	B	For SARI patients, VE against influenza B was 76% (95% CI: 54 to 87); For ILI patients, VE against influenza B was 54% (95% CI: 19 to 75)	M	(16)
2016	A(H3N2)	4% (-40-36%)	U	(17)
2018	A(H3N2)	25% (13-36%)†	U	Uses NH vaccine effectiveness value for 2017/2018 period (14)

* Values shown represent VE against all subtypes. †No vaccine effectiveness (VE) values available for this period, as such the VE values for the preceding NH vaccine are used

4 Further information on the economic evaluation

4.1 Economic evaluation decision tree

We used an economic evaluation decision tree to categorise infected individuals as asymptomatic, symptomatic with mild illness (upper respiratory tract (URT) infections) or symptomatic with severe illness (lower respiratory tract (LRT) infections) based on published data from influenza challenge studies (8). Those with mild illness were either seen at an outpatient clinic or were not medically attended, while patients with severe illness were either hospitalised or not. All those with mild illness were assumed to recover, while those with severe illness either recovered or died. The values of the disease states, and healthcare utilisation events associated with each stage are presented in the main text.

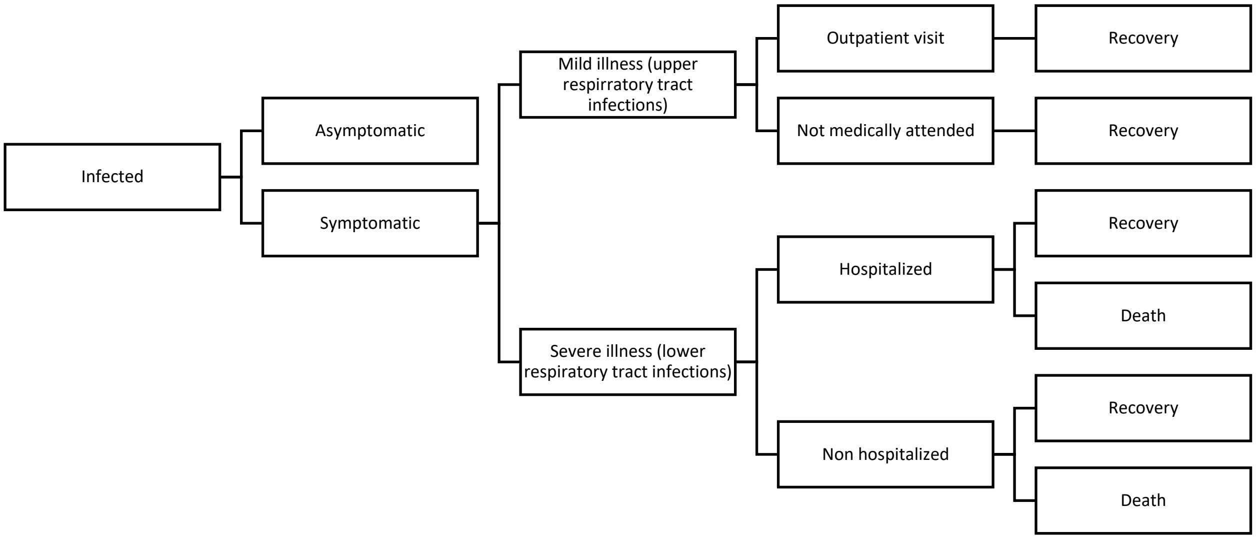

Additional file 2 figure 4: Economic evaluation decision tree of influenza infection and healthcare utilization

4.2 Additional inputs in the economic model

Additional file 2 table 6: Gross domestic product (GDP) per capita values, GDP deflator values and currency exchange rated

Input	Value	Source
**Gross Domestic Product (GDP) per capita**		
GDP per capita 2018	1,710.5	(18)
**GDP deflator**		
2018 GDP deflator	192.255	(19)
2014 GDP deflator	140.613	
2012 GDP deflator	123.721	
**Kenya shilling to US dollar exchange rate**		
2017 exchange rate for one dollar	103.2317 KES	(20)
2014 exchange rate for one dollar	90 KES	(21)
2012 exchange rate for one dollar	83 KES	(22)

KES – Kenya shillings

Additional file 2 table 7: Life expectancy values used in calculation of disability adjusted life years (DALYs) that were obtained from the Global Health Observatory data repository (23)

Life expectancy	**2018***	**2017***	**2016**	**2015**	**2014**	**2013**	**2012**	**2011**	**2010**
<1 year	66.2	66.1	66.7	66.0	65.6	65.3	64.9	64.0	62.9
1-4 years	67.8	67.7	68.2	67.7	67.2	67.0	66.7	65.7	64.6
10-14 years	60.3	60.1	60.6	60.1	59.8	59.6	59.4	58.5	57.5
15-19 years	55.6	55.5	55.9	55.4	55.1	55.0	54.8	53.9	53.0
30-34 years	42.5	42.4	42.8	42.3	42.1	41.9	41.7	41.0	40.1
70-74 years	11.7	11.7	11.7	11.7	11.7	11.7	11.6	11.6	11.4

*Estimated value based on average of three previous years

Additional file 2 table 8: Disability adjusted life year (DALY) weights used in economic model obtained from the Global Burden of Disease Study, 2016 (24)

DALY weights	Value	Additional notes
Influenza cases with mild illness/upper respiratory tract infection	0.006 (0.002-0.012)	Disability weight for mild upper respiratory infection is used
Influenza cases with lower respiratory tract illness that are not hospitalised	0.051 (0.032-0.074)	Disability weight for moderate lower respiratory infection is used
Influenza cases with lower respiratory tract illness that are hospitalised	0.133 (0.088-0.19)	Disability weight for severe lower respiratory infections is used

5 Further information on fitted model

5.1 Fitted periods of influenza activity

We identified periods of high influenza activity as >2 successive weeks where the proportion of subtype-specific test-positive cases was greater than the average weekly proportion during the entire study. A period ended when there were ≥2 consecutive weeks where the proportion of subtype-specific positive cases was less than the weekly average. In addition, influenza-positive cases had to be observed in at least 3 of the 5 surveillance sites so that periods identified were of widespread transmission. Periods were included if the posterior mean estimate of the net reproduction number at the start of the simulation was greater than or equal to 1.

There were 4 peaks in influenza B activity, 3 peaks in influenza A(H3N2) activity and 2 peaks in influenza A(H1N1)pdm09 activity. Influenza A(H1N1)pdm09 data from January 2010 to December 2011 was excluded from the analysis as this coincided with the emergence of the pandemic A(H1N1)pdm09 virus.

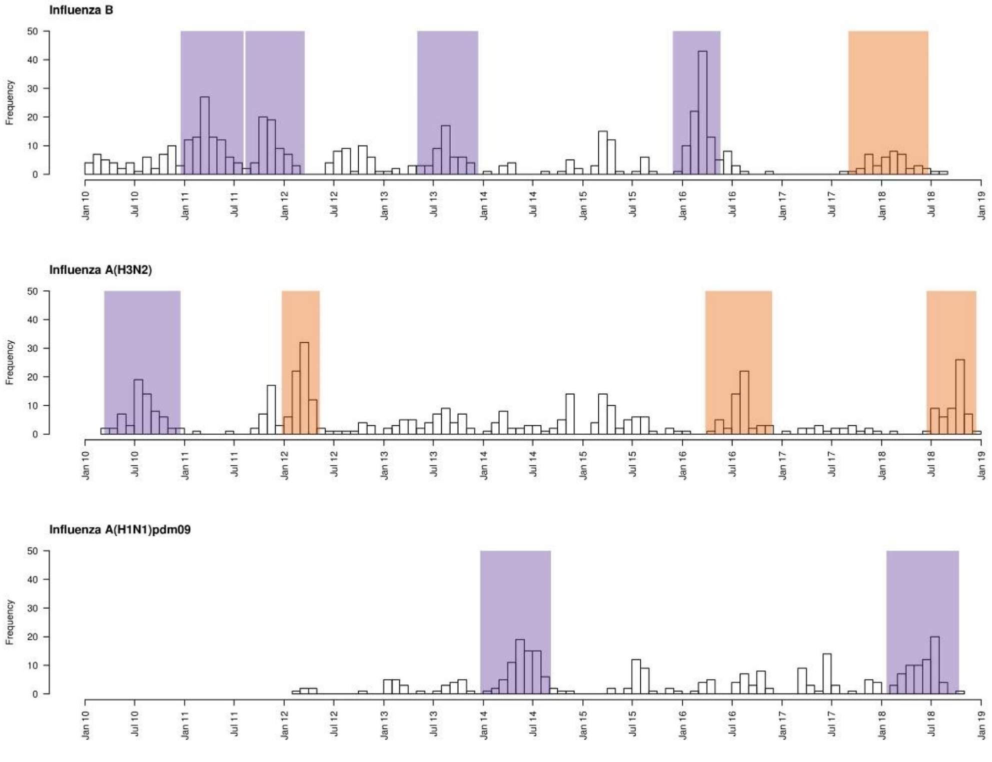

Additional file 2 figure 5: Epidemic curve of modelled peaks in influenza activity by influenza subtype and vaccine effectiveness. Shaded area refers to the identified peaks in influenza activity. Purple shading refers to seasons where the vaccine was well matched to the circulating strains (vaccine effectiveness (VE) = 70%). Orange shading refers to seasons where the vaccine was poorly matched to circulating strains (VE = 42%). There was no influenza activity detected between September 2014-August 2015 and September 2016-August 2017. There was no Southern Hemisphere VE data available for the A(H3N2) season in June 2018-December 2018, so the Northern Hemisphere VE data for the 2017 to 2018 period was used.

5.2 Periods that did not meet decision rule criteria

The periods listed below had >2 successive weeks where the proportion of subtype-specific test-positive cases was greater than the average weekly proportion during the entire study, however, they did not meet the decision rule criteria because either transmission was recorded in less than 3 of the surveillance sites or the mean net reproduction number at the start of the period was less than 1.

Additional file 2 table 9: Periods that did not meet decision rule criteria

Period	Flu type/subtype
Jan 2010 to Dec 2011	A H1N1pdm09
Oct 2011 to Dec 2011	A H3N2
Jun 2012 to Sep 2012	B
Sep 2012 to Dec 2012	B
Jan 2015 to Jun 2015	B
Feb 2015 to May 2015	A H3N2
Jun 2015 to Sep 2015	A H1N1pdm09
Sep 2017 to Jun 2018	B

5.3 Fit of model to data and distribution of posteriors

5.3.1 Influenza B

5.3.1.1 17 September 2010 to 05 August 2011

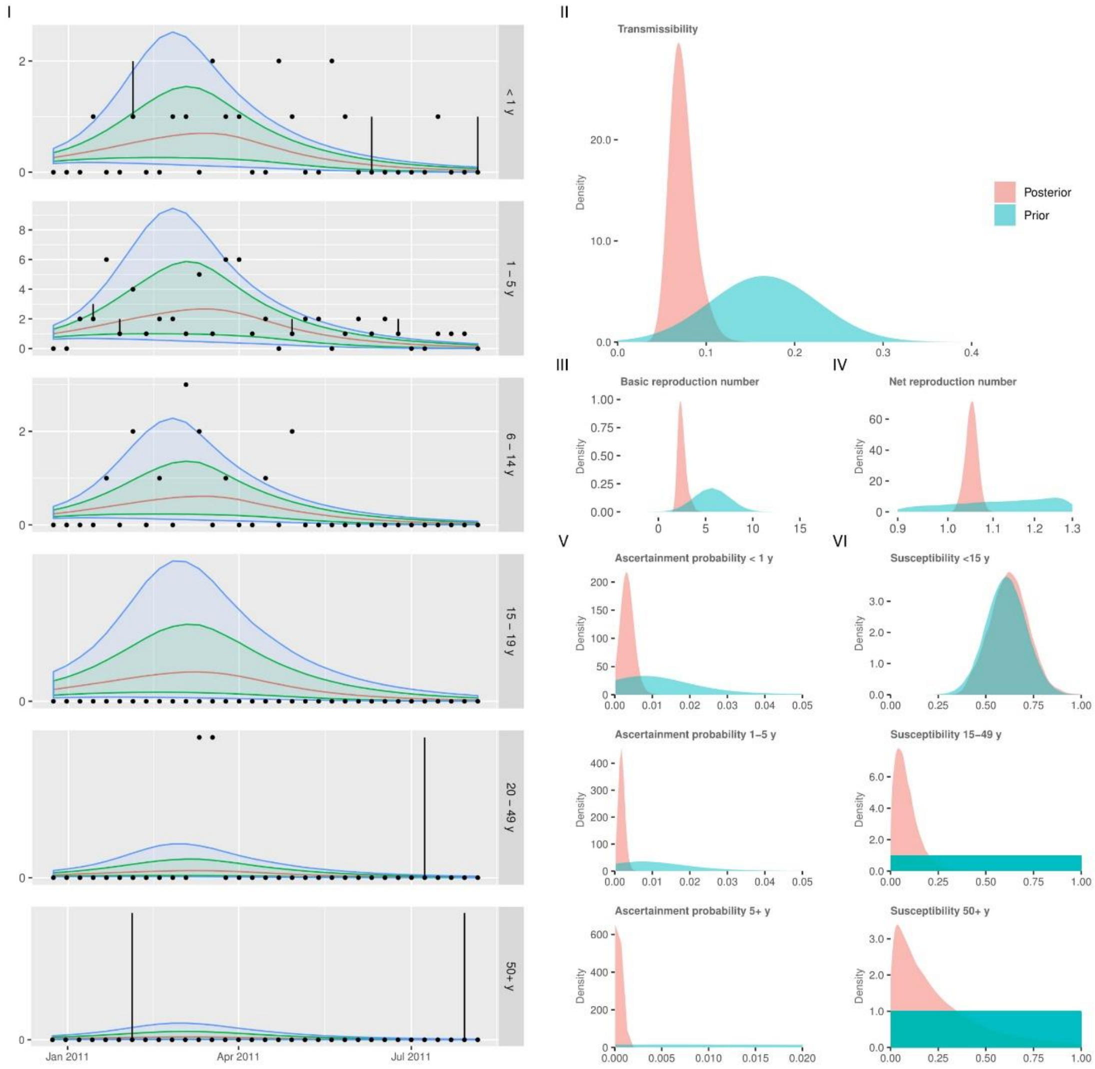

Additional file 2 figure 6: Inference results for influenza B activity, September 2010 to August 2011.
I. *Comparison of the fit of the model to the age specific time series of influenza positive SARI cases detected in the influenza surveillance system (black dots) with hypergeometric 95% confidence interval. The median (red) and 50 and 75% credible intervals (shaded green and blue respectively) from the fitted model. II. Transmissibility of the virus. III. Basic reproduction number. IV. Net reproduction number. V. Ascertainment probability in 3 age groups. VI. Susceptibility in 3 age groups. For II to VI the prior distributions are in blue and posterior outputs in pink.*

5.3.1.2 12 August 2011 to 16 March 2012

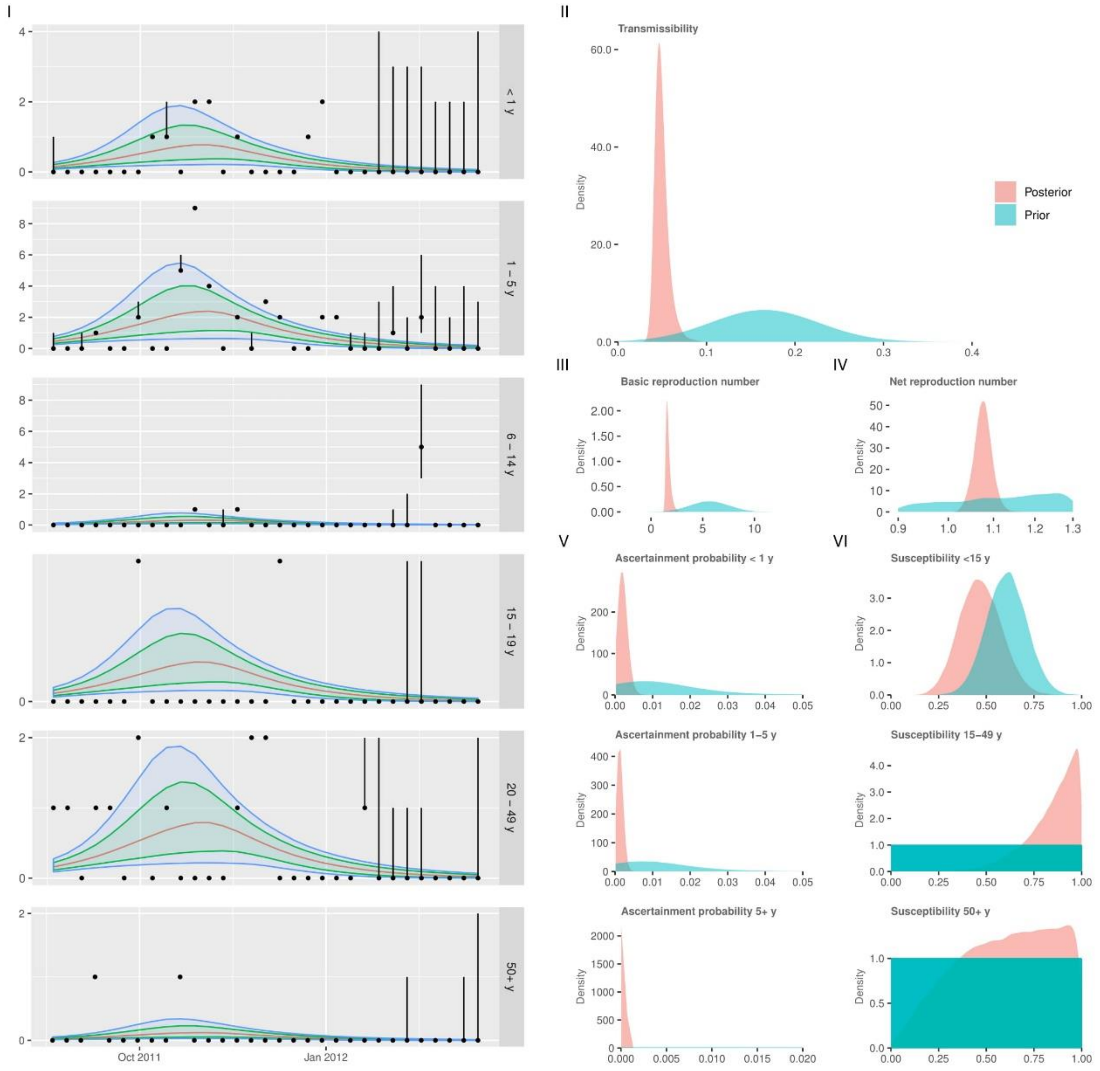

Additional file 2 figure 7: Inference results for influenza B activity, August 2011 to March 2012
I. *Comparison of the fit of the model to the age specific time series of influenza positive SARI cases detected in the influenza surveillance system (black dots) with hypergeometric 95% confidence interval. The median (red) and 50 and 75% credible intervals (shaded green and blue respectively) from the fitted model. II. Transmissibility of the virus. III. Basic reproduction number. IV. Net reproduction number. V. Ascertainment probability in 3 age groups. VI. Susceptibility in 3 age groups. For II to VI the prior distributions are in blue and posterior outputs in pink.*

5.3.1.3 3 May 2013 to 13 December 2013

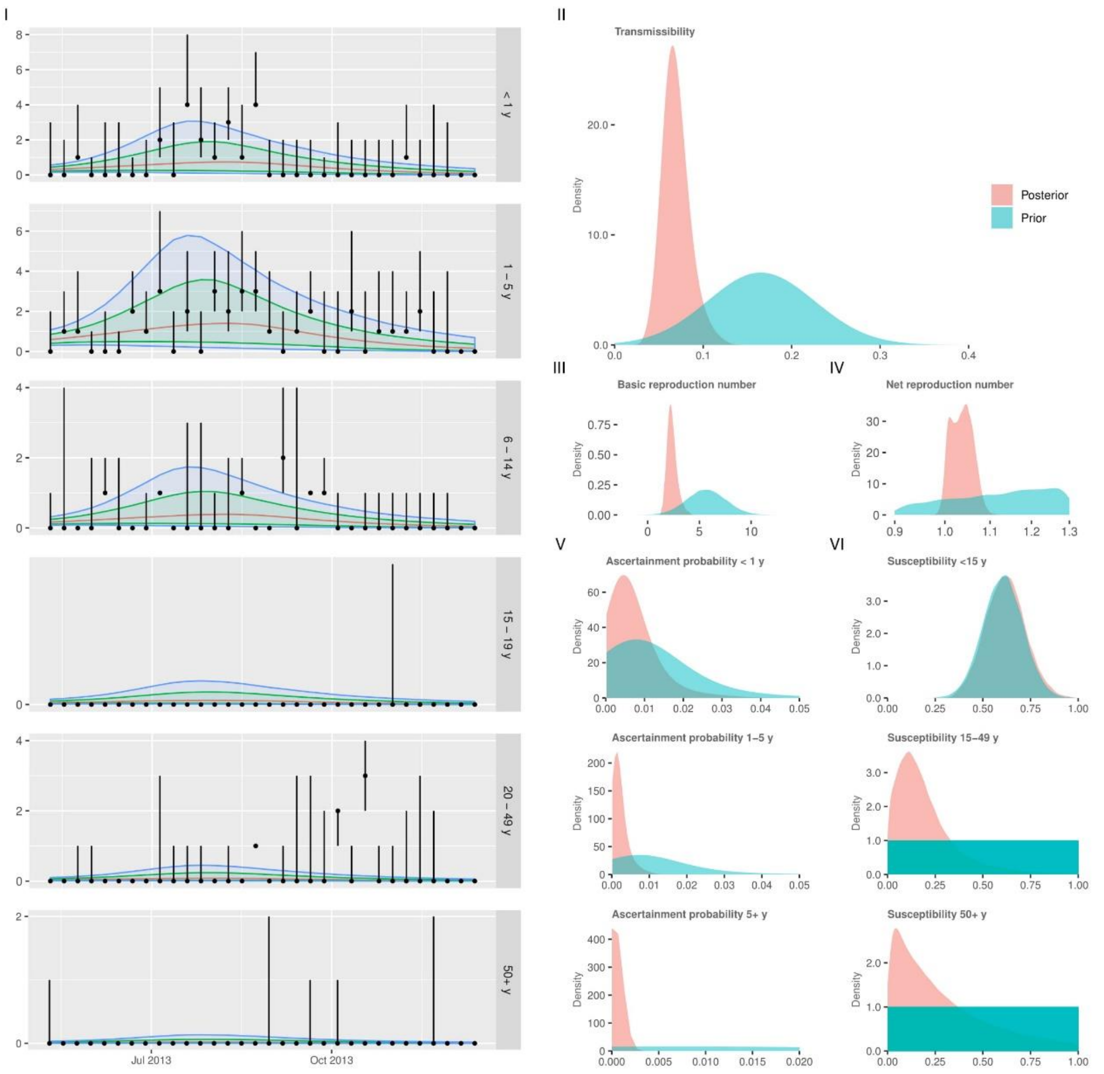

Additional file 2 figure 8: Inference results for influenza B activity, May to December 2013
I. *Comparison of the fit of the model to the age specific time series of influenza positive SARI cases detected in the influenza surveillance system (black dots) with hypergeometric 95% confidence interval. The median (red) and 50 and 75% credible intervals (shaded green and blue respectively) from the fitted model. II. Transmissibility of the virus. III. Basic reproduction number. IV. Net reproduction number. V. Ascertainment probability in 3 age groups. VI. Susceptibility in 3 age groups. For II to VI the prior distributions are in blue and posterior outputs in pink.*

5.3.1.4 27 November 2015 to 20 May 2016

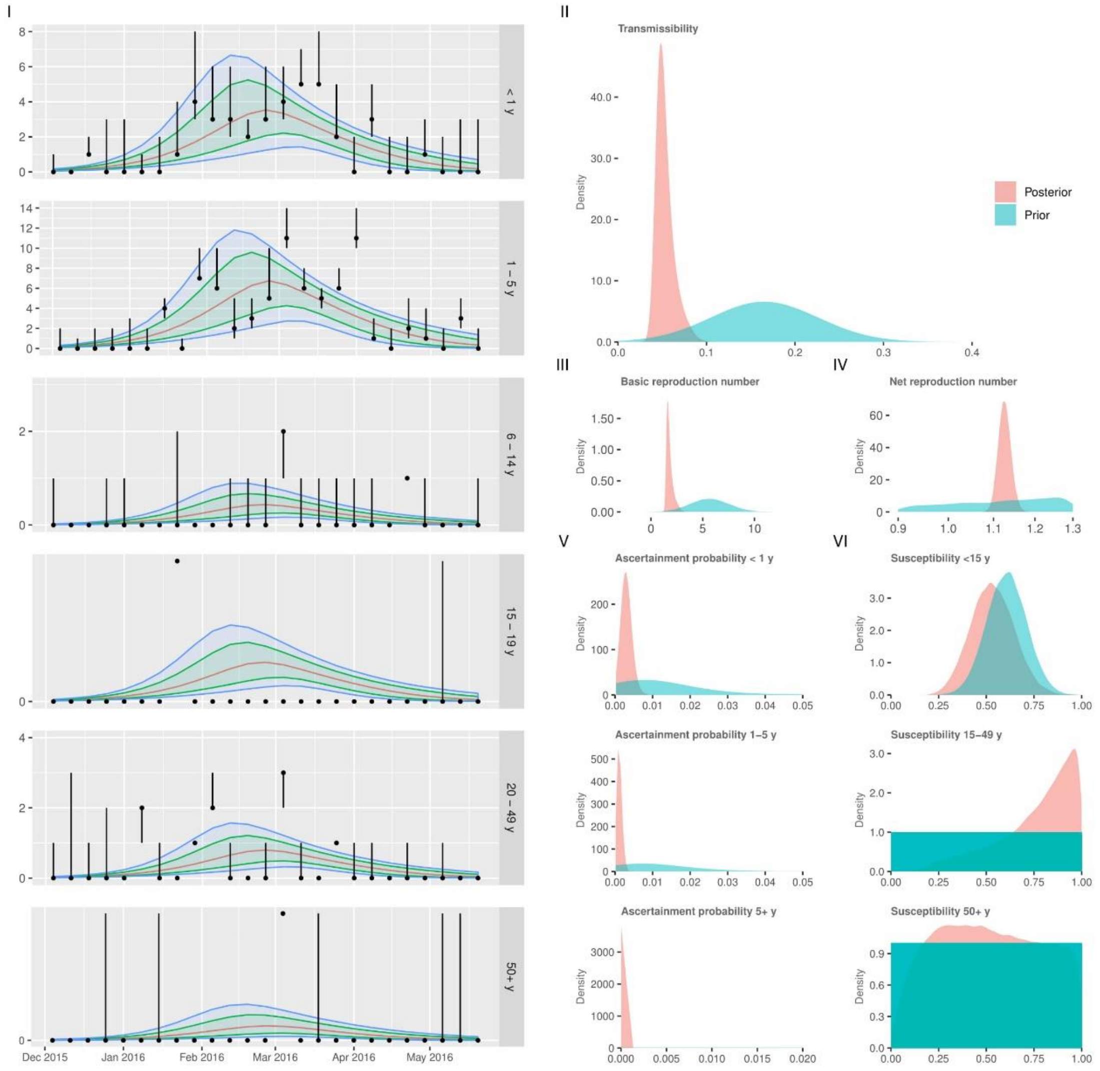

Additional file 2 figure 9: Inference results for influenza B activity, November 2015 to May 2016
I. *Comparison of the fit of the model to the age specific time series of influenza positive SARI cases detected in the influenza surveillance system (black dots) with hypergeometric 95% confidence interval. The median (red) and 50 and 75% credible intervals (shaded green and blue respectively) from the fitted model. II. Transmissibility of the virus. III. Basic reproduction number. IV. Net reproduction number. V. Ascertainment probability in 3 age groups. VI. Susceptibility in 3 age groups. For II to VI the prior distributions are in blue and posterior outputs in pink.*

5.3.1.5 1 September 2017 to 22 June 2018

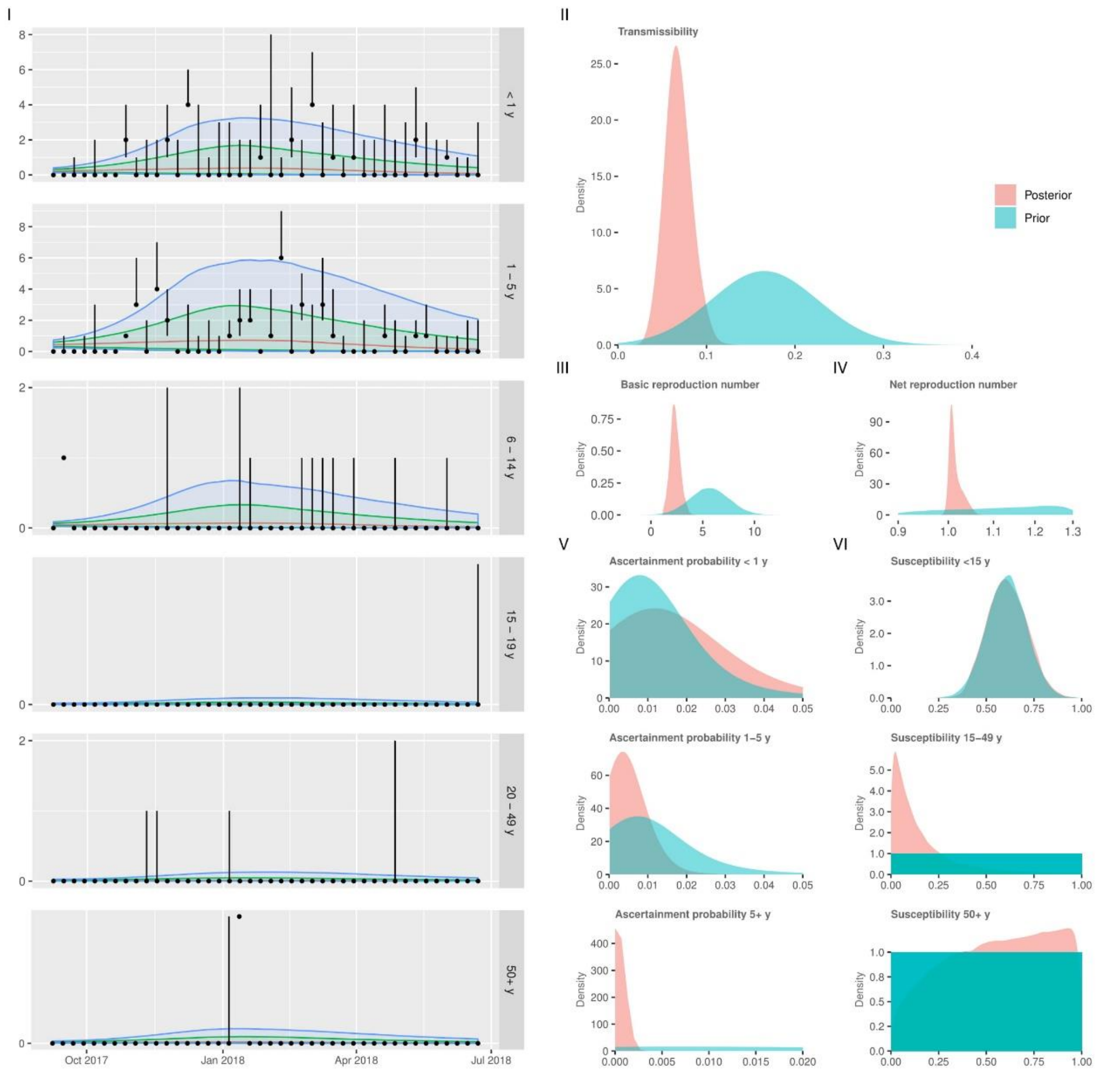

Additional file 2 figure 10: Inference results on influenza B activity, September 2017 to June 2018
I. *Comparison of the fit of the model to the age specific time series of influenza positive SARI cases detected in the influenza surveillance system (black dots) with hypergeometric 95% confidence interval. The median (red) and 50 and 75% credible intervals (shaded green and blue respectively) from the fitted model. II. Transmissibility of the virus. III. Basic reproduction number. IV. Net reproduction number. V. Ascertainment probability in 3 age groups. VI. Susceptibility in 3 age groups. For II to VI the prior distributions are in blue and posterior outputs in pink.*

5.3.2 Influenza A(H3N2)

5.3.2.1 12 March 2010 to 17 December 2010

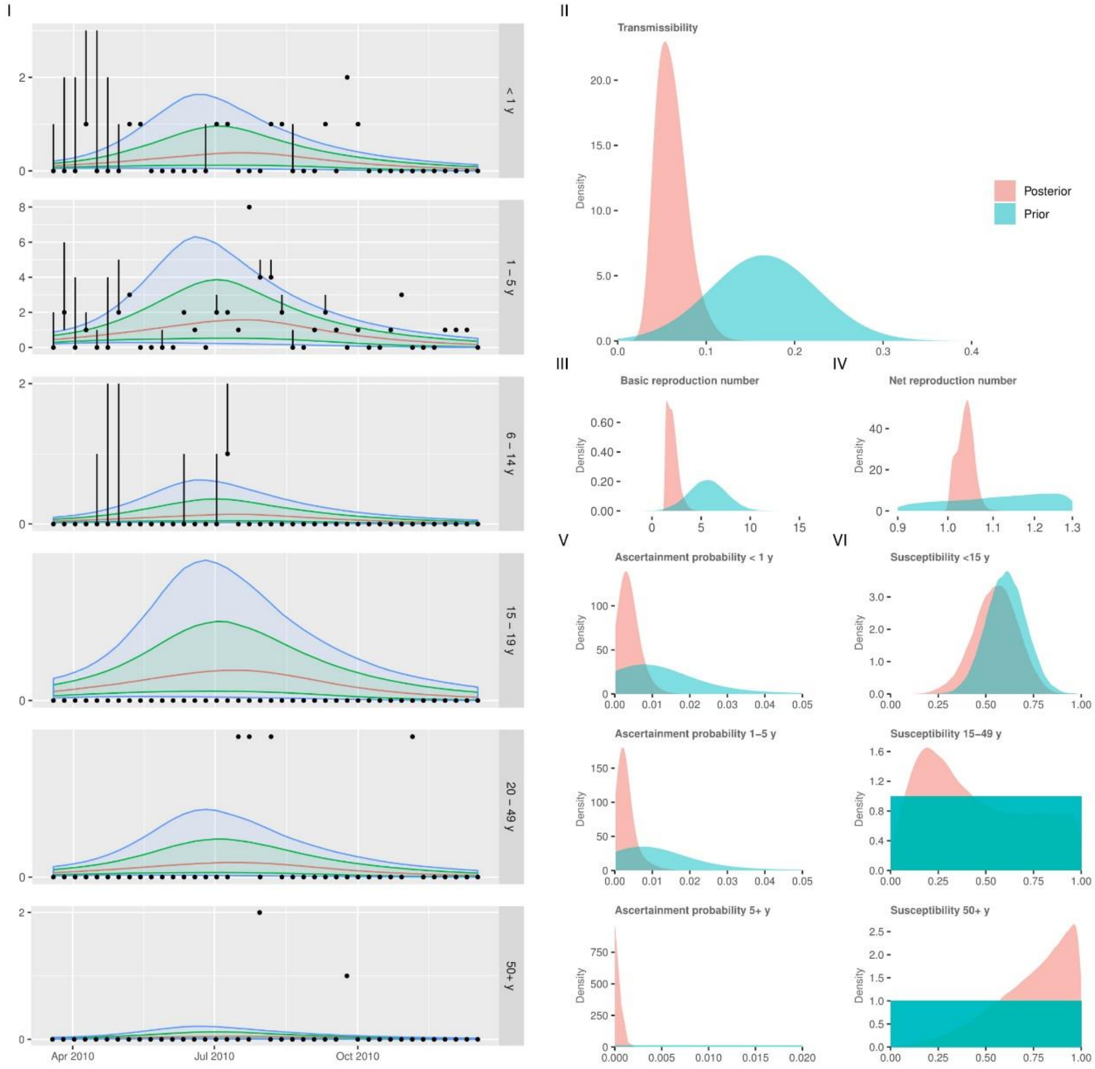

Additional file 2 figure 11: Inference results for influenza A(H3N2) activity, March to December 2010
I. *Comparison of the fit of the model to the age specific time series of influenza positive SARI cases detected in the influenza surveillance system (black dots) with hypergeometric 95% confidence interval. The median (red) and 50 and 75% credible intervals (shaded green and blue respectively) from the fitted model. II. Transmissibility of the virus. III. Basic reproduction number. IV. Net reproduction number. V. Ascertainment probability in 3 age groups. VI. Susceptibility in 3 age groups. For II to VI the prior distributions are in blue and posterior outputs in pink.*

5.3.2.2 23 December 2011 to 11 May 2012

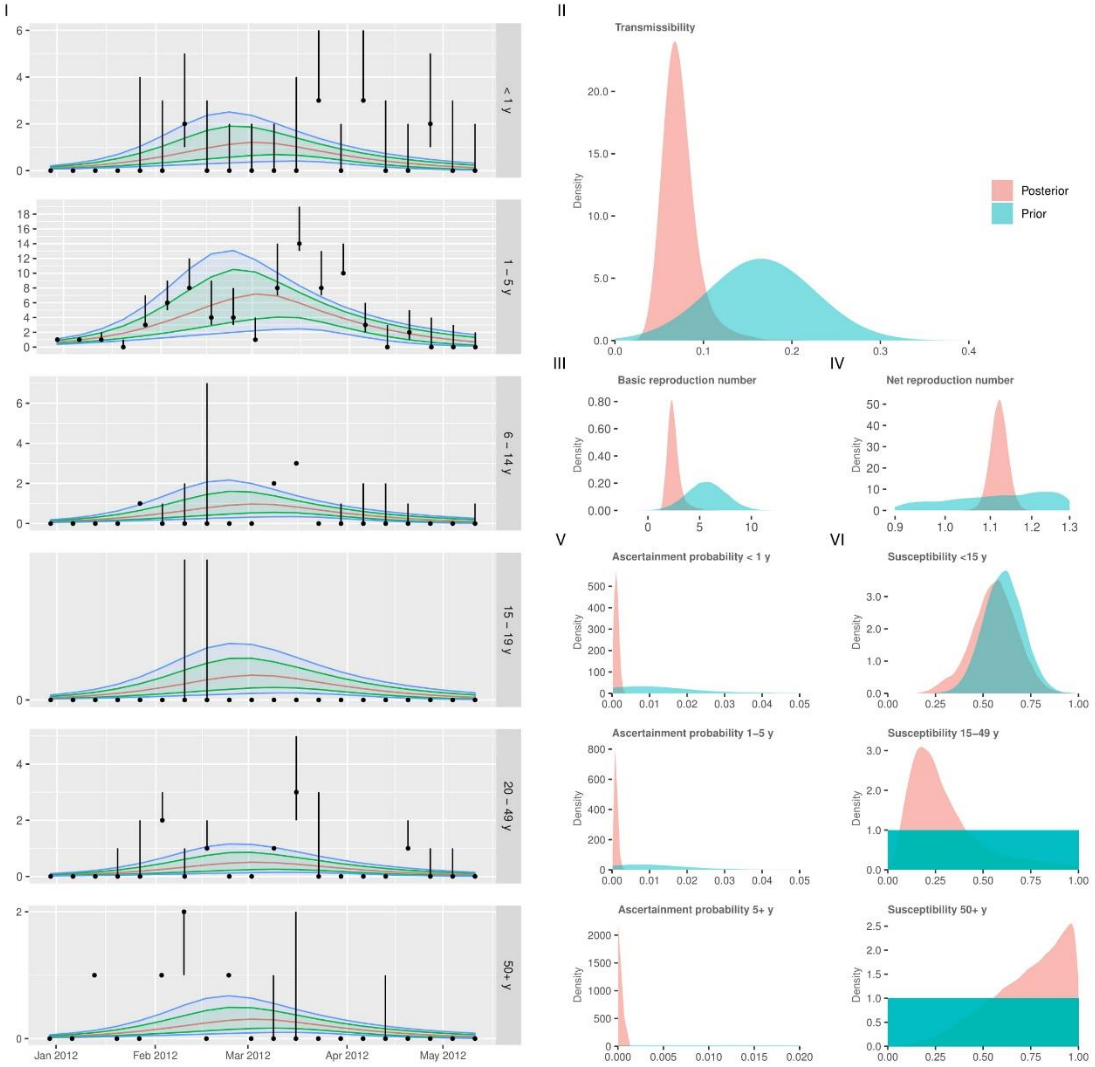

Additional file 2 figure 12: Inference results for influenza A(H3N2) activity, December 2011 to May 2012
I. *Comparison of the fit of the model to the age specific time series of influenza positive SARI cases detected in the influenza surveillance system (black dots) with hypergeometric 95% confidence interval. The median (red) and 50 and 75% credible intervals (shaded green and blue respectively) from the fitted model. II. Transmissibility of the virus. III. Basic reproduction number. IV. Net reproduction number. V. Ascertainment probability in 3 age groups. VI. Susceptibility in 3 age groups. For II to VI the prior distributions are in blue and posterior outputs in pink.*

5.3.2.3 25 March 2016 to 25 November 2016

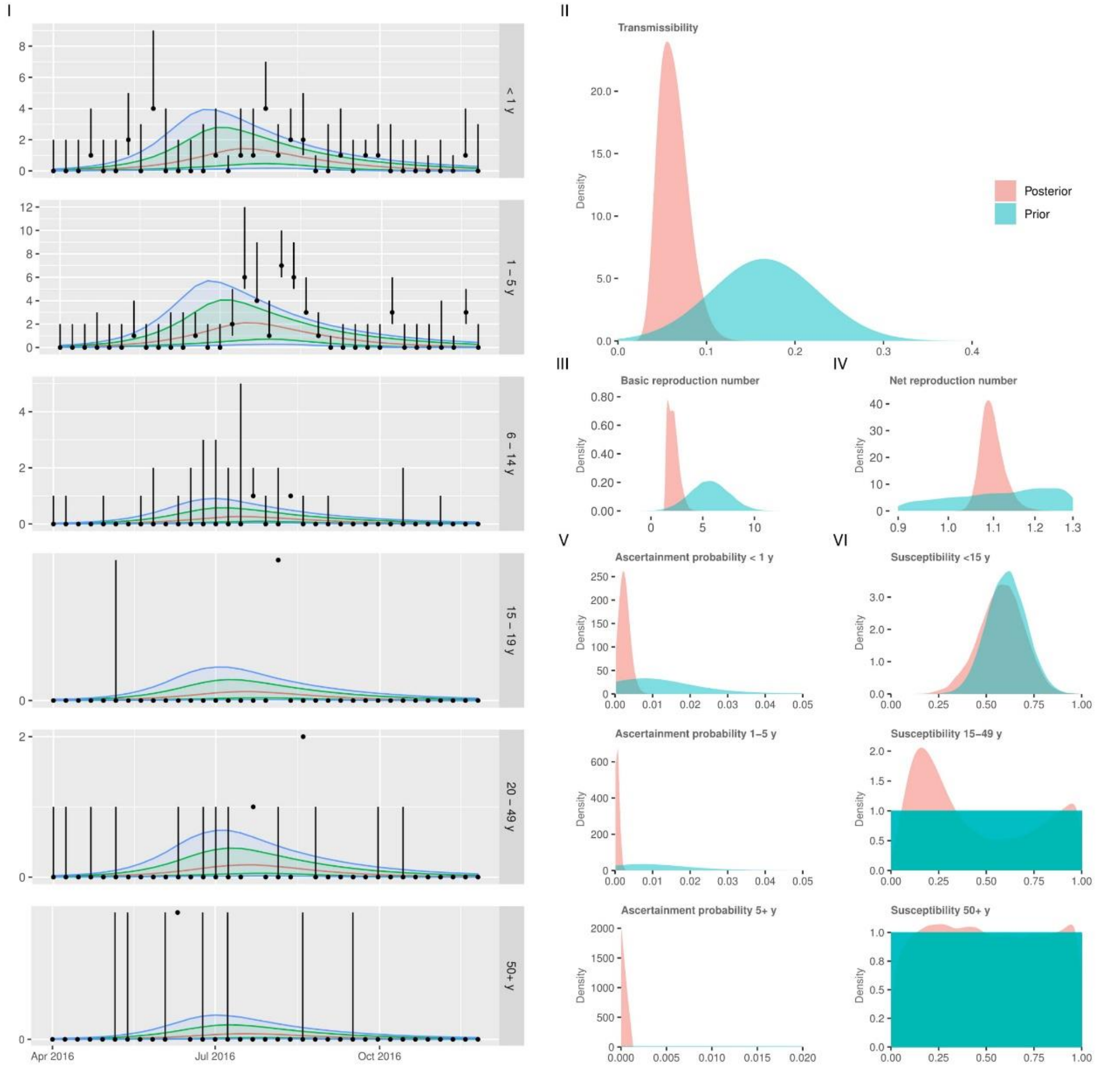

Additional file 2 figure 13: Inference results for influenza A(H3N2) activity, March to November 2016
I. *Comparison of the fit of the model to the age specific time series of influenza positive SARI cases detected in the influenza surveillance system (black dots) with hypergeometric 95% confidence interval. The median (red) and 50 and 75% credible intervals (shaded green and blue respectively) from the fitted model. II. Transmissibility of the virus. III. Basic reproduction number. IV. Net reproduction number. V. Ascertainment probability in 3 age groups. VI. Susceptibility in 3 age groups. For II to VI the prior distributions are in blue and posterior outputs in pink.*

5.3.2.4 *15 June 2018 to 14 December 2018*

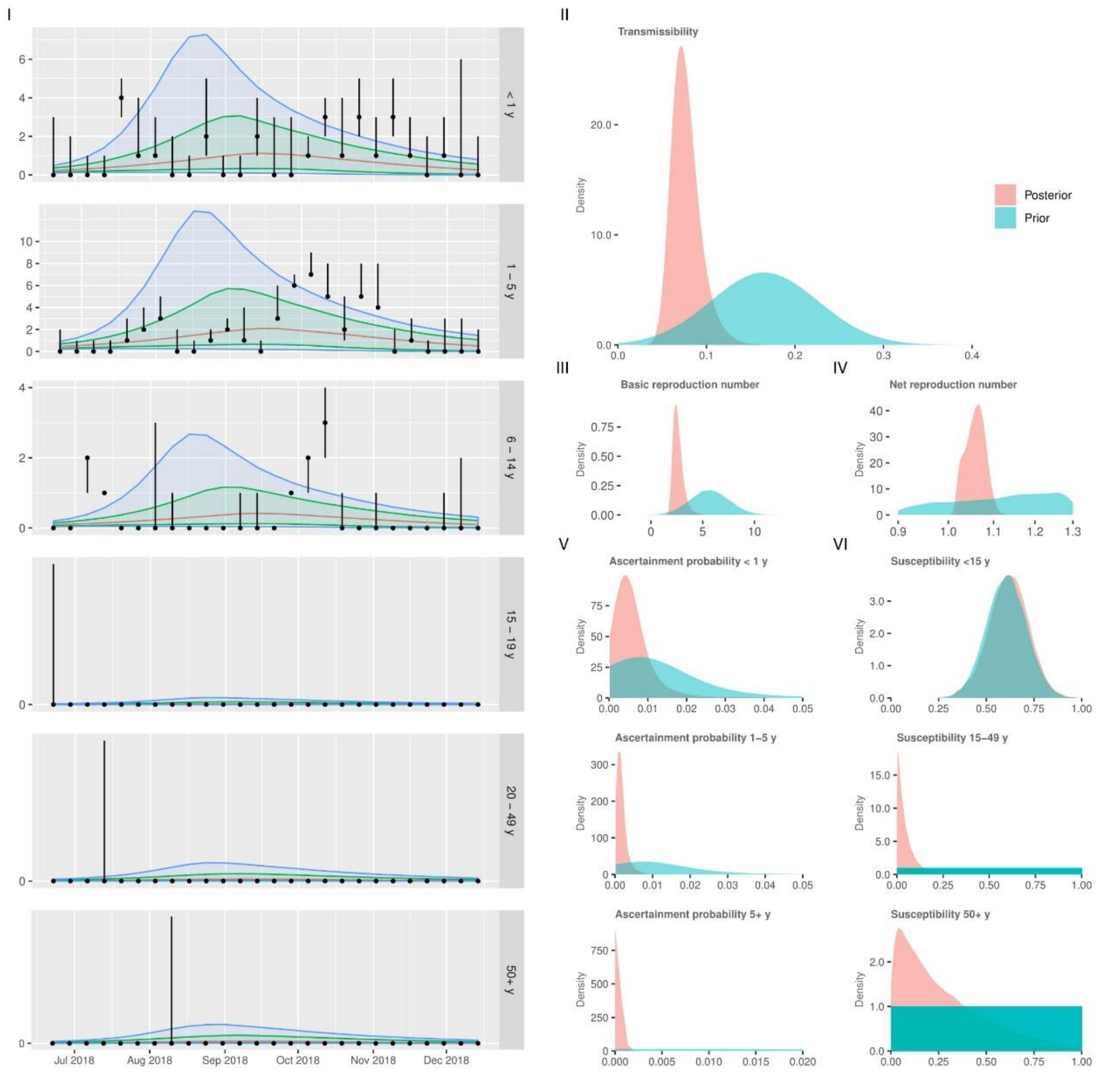

Additional file 2 figure 14: Inference results for influenza A(H3N2) activity, June to December 2018
I. Comparison of the fit of the model to the age specific time series of influenza positive SARI cases detected in the influenza surveillance system (black dots) with hypergeometric 95% confidence interval. The median (red) and 50 and 75% credible intervals (shaded green and blue respectively) from the fitted model. II. Transmissibility of the virus. III. Basic reproduction number. IV. Net reproduction number. V. Ascertainment probability in 3 age groups. VI. Susceptibility in 3 age groups. For II to VI the prior distributions are in blue and posterior outputs in pink.

5.3.3 Influenza A(H1N1)pdm09

5.3.3.1 20 December 2013 to 5 September 2014

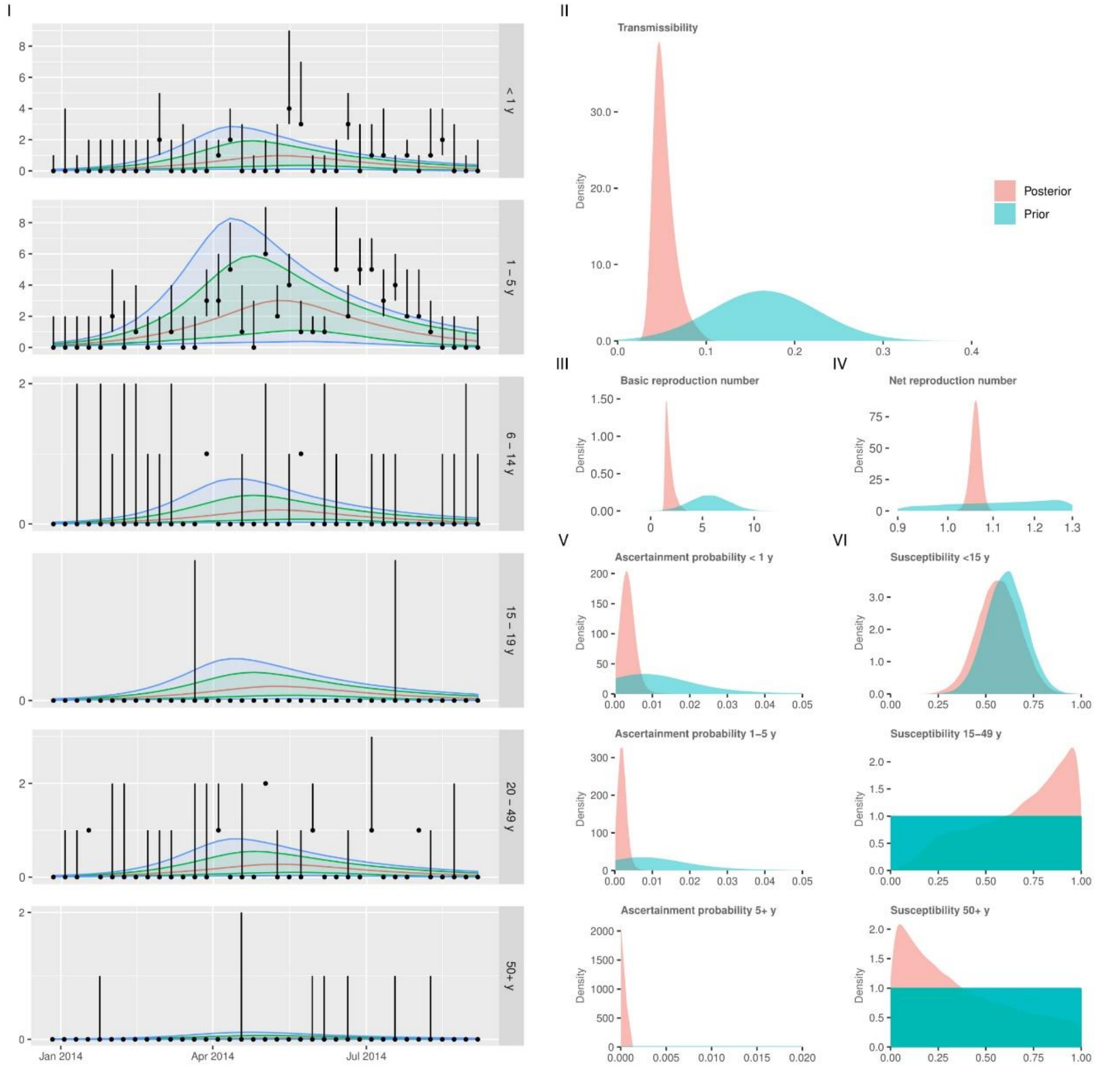

Additional file 2 figure 15: Inference results for influenza A(H1N1)pdm09 activity, December 2013 to September 2014
I. *Comparison of the fit of the model to the age specific time series of influenza positive SARI cases detected in the influenza surveillance system (black dots) with hypergeometric 95% confidence interval. The median (red) and 50 and 75% credible intervals (shaded green and blue respectively) from the fitted model. II. Transmissibility of the virus. III. Basic reproduction number. IV. Net reproduction number. V. Ascertainment probability in 3 age groups. VI. Susceptibility in 3 age groups. For II to VI the prior distributions are in blue and posterior outputs in pink.*

5.3.3.2 19 January 2018 to 12 October 2018

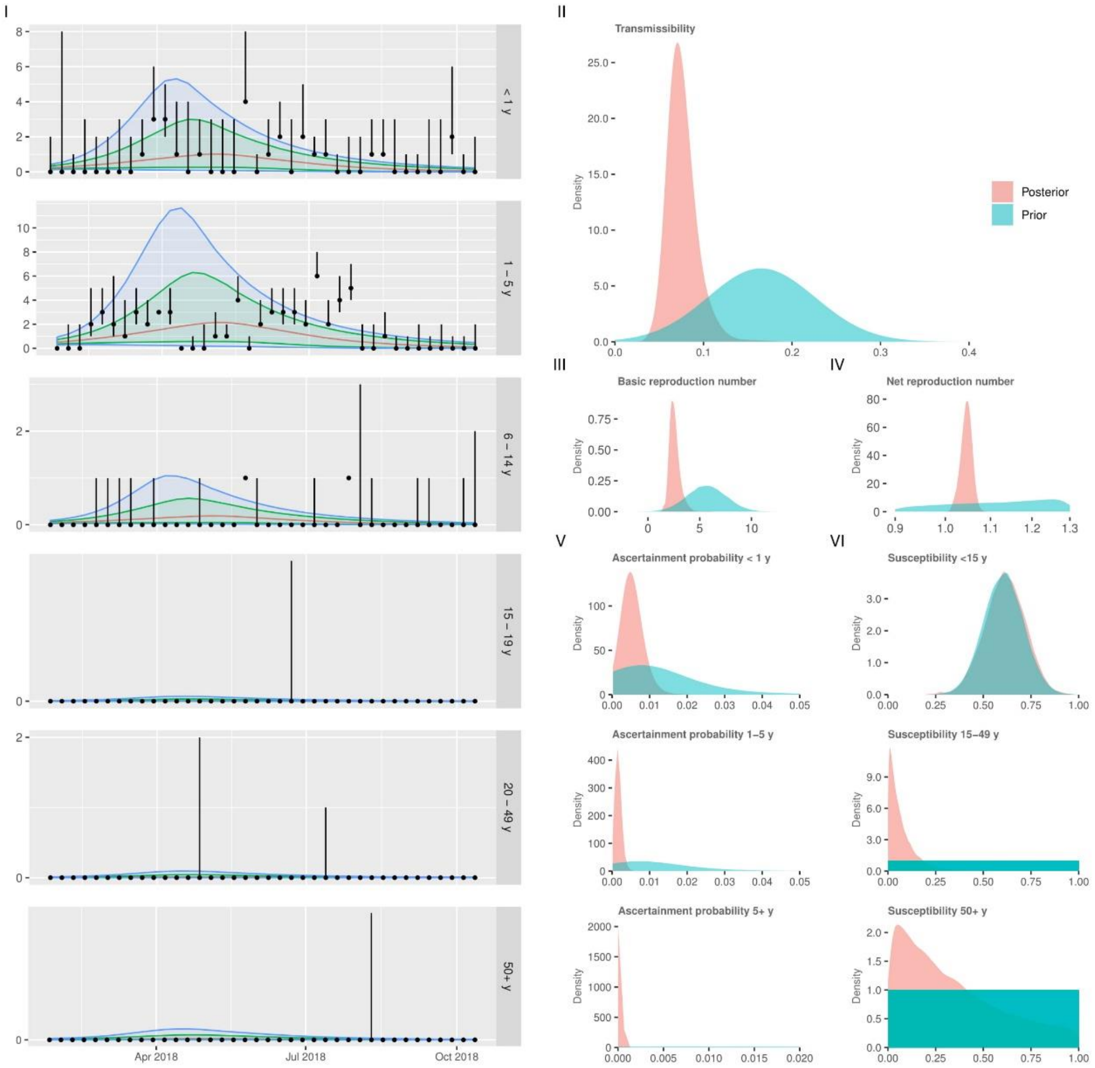

Additional file 2 figure 16: Inference results for influenza A(H1N1)pdm09 activity, January to October 2018
I. *Comparison of the fit of the model to the age specific time series of influenza positive SARI cases detected in the influenza surveillance system (black dots) with hypergeometric 95% confidence interval. The median (red) and 50 and 75% credible intervals (shaded green and blue respectively) from the fitted model. II. Transmissibility of the virus. III. Basic reproduction number. IV. Net reproduction number. V. Ascertainment probability in 3 age groups. VI. Susceptibility in 3 age groups. For II to VI the prior distributions are in blue and posterior outputs in pink.*

6 Additional results

This section provides additional information on the incremental net monetary benefits (INMB) obtained for each strategy using total societal costs and direct medical costs only, as well as the ICER values obtained from the sensitivity analysis.

6.1 Results of incremental net monetary benefit analysis

Additional file 2 table 10: Annual willingness-to-pay threshold values at which influenza vaccination was cost-effective using total societal costs and direct medical costs only

**Year**	**2010**	**2010-2011**	**2011-2012**	**2012-2013**	**2013-2014**	**2015-2016**	**2017-2018**
WTP USD value at which vaccination resulted in a positive INMB value using **total societal costs**	$428	$736	NA*	$511	$428	$478	$246
WTP USD value at which vaccination resulted in a positive INMB value **using direct medical costs only**	$574	$901	NA*	$687	$581	$639	$441
Most optimal strategy at the WTP value	IA	IB	NA*	IA	IB	IIIA	IB

*INMB – incremental net monetary benefit; WTP – willingness-to-pay; USD – US dollar. *In this year, vaccination was not cost effective using a WTP threshold of $18-872 per DALY averted*

Additional file 2 table 11: Vaccination strategy with the highest positive incremental net monetary benefit in the year using costs calculated from total societal costs and direct medical costs

**Year**	Willingness-to-pay threshold using **total societal costs**			Willingness-to-pay threshold using **direct medical costs** only		
	Minimum value $17	Median value $445	Maximum value $872	Minimum value $17	Median value $445	Maximum value $872
**2010**	None	IA	IIA	None	None	IIA
**2010-2011**	None	None	IIC	None	None	None
**2011-2012**	None	None	None	None	None	None
**2012-2013**	None	None	IIA	None	None	IIA
**2013-2014**	None	IB	IIIB	None	None	IIIB
**2015-2016**	None	None	IIIC	None	None	IIIA
**2017-2018**	None	IIB	IIIC	None	IB	IIIC

6.2 Results of sensitivity analysis

During one-way sensitivity analysis, strategy IB (vaccinating children 6-23 months of age between October-December) remained the most cost-effective strategy i.e. attained the highest INMB value at the lowest willingness-to-pay WTP value. However, no vaccination strategy was cost effective at the upper limit of the WTP threshold ($872) when vaccine price was increased to $4.5, $6 and $10. The following sections describe the ICER values obtained during sensitivity analysis.

6.2.1 Social weighting and time discounting

During the sensitivity analysis we maintained the vaccine purchase price at $3.0 per dose and calculated DALYs with and without social weighting and time discounting. Social weighting placed greater value on life lost from 9-56 years of age. Removing time discounting led to a 49-50% reduction in mean ICER per DALY averted across all strategies, and addition of social weighting led to a slight decrease (5-6%) in mean ICER value.

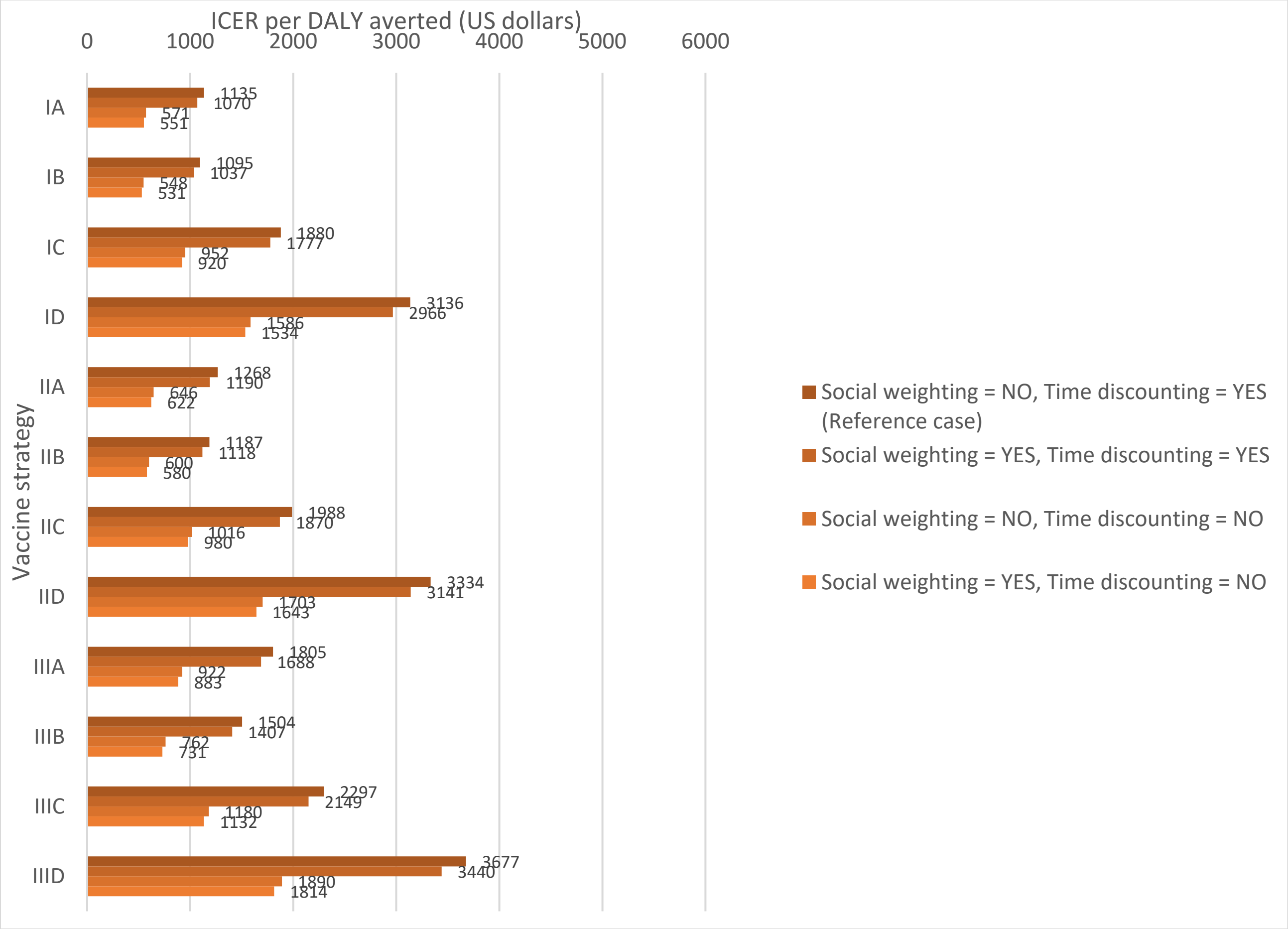

Additional file 2 figure 17: Mean annual ICER values per DALY averted per strategy with and without time discounting and social weighting. Strategies are vaccinating children 6-23 months (strategy I), 2-5 years (strategy II) and 6-14 years (strategy III) with

either the Southern Hemisphere influenza vaccine (Strategy A) or Northern Hemisphere vaccine (Strategy B) or both (Strategy C: twice yearly 3-month vaccination periods, or Strategy D: year-round vaccination).

6.2.2 Changes in vaccine price

In the reference case we calculated the ICER per DALY averted with time discounting at 3% and no social weighting. During sensitivity analysis we varied the vaccine purchase price per dose to be $1.5, $4.5, $6.0 and $10.0. At a vaccine purchase price of $10.0 USD the mean ICER value increased by 144-178%, at $6.0 USD the mean ICER value increased by 38-43%, while at a vaccine purchase price of $4.5 USD the mean ICER value increased by 31-38%. At a vaccine purchase price of $1.5 USD the mean ICER decreased by 44-62%.

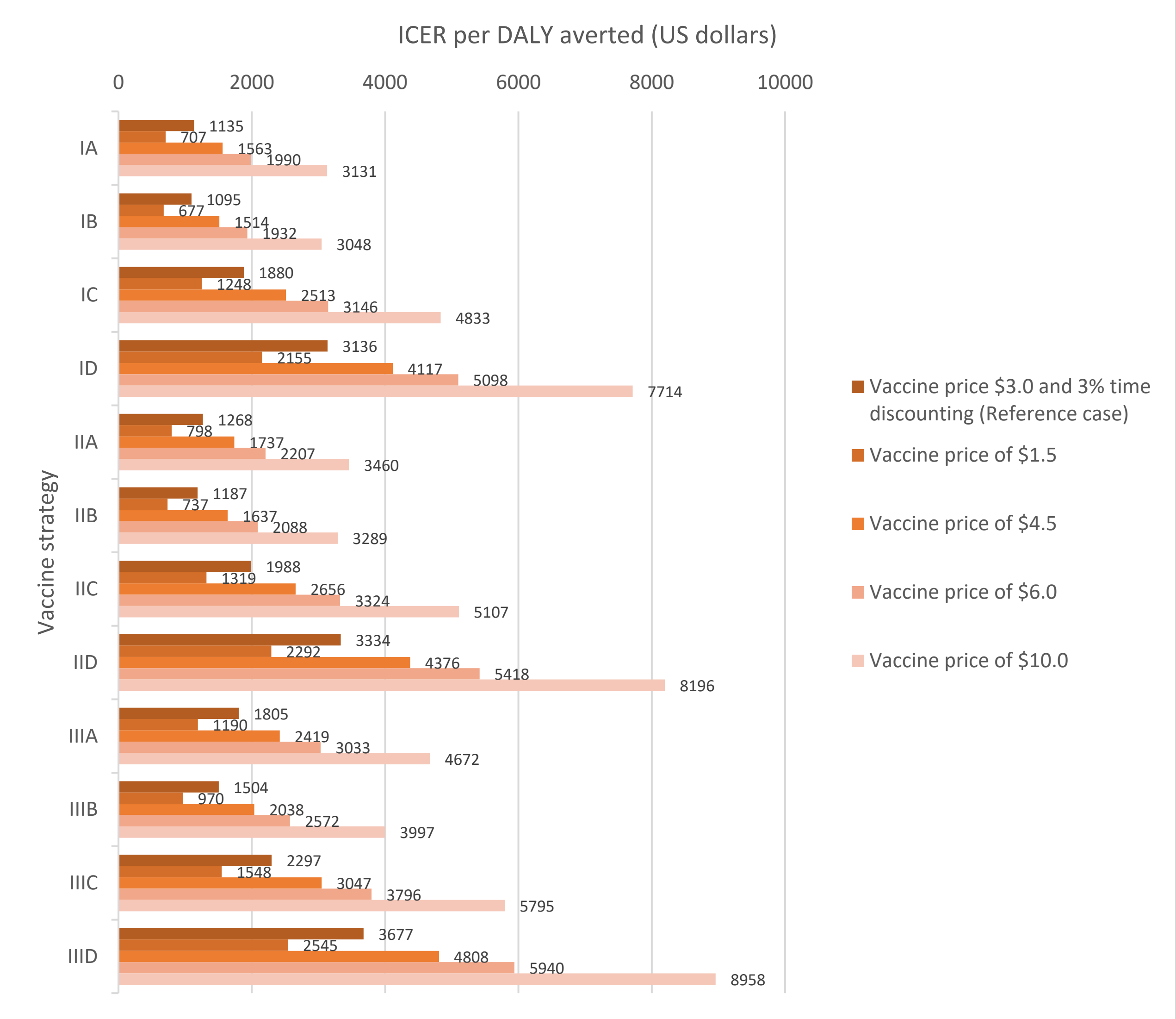

Additional file 2 figure 18: Mean annual ICER values per DALY averted per strategy at different vaccine prices. Strategies are vaccinating children 6-23 months (strategy I), 2-5 years (strategy II) and 6-14 years (strategy III) with either the Southern Hemisphere influenza vaccine (Strategy A) or Northern Hemisphere vaccine (Strategy B) or both (Strategy C: twice yearly 3-month vaccination periods, or Strategy D: year-round vaccination).

6.2.3 Changes in vaccine coverage

We assessed the impact of maintaining vaccination coverage across all age groups at the same level of coverage attained in strategy I i.e. 30% for once yearly vaccination, 45% for twice yearly vaccination and 60% for year-round vaccination. The mean ICER value decreased by 1-4% for II strategies and 7-20% for III.

7 Comparison between UK model and Kenya model

This section compares key inputs and outputs of the transmission model for the original paper (5, 6) fitted to UK surveillance data, and the adaption to Kenya.

Difference	United Kingdom	Kenya	Impact
Epidemic timing	Well defined annual peak of influenza activity in Northern Hemisphere season	No defined primary peak in influenza activity. Equal activity in Northern Hemisphere and Southern Hemisphere season. Significant year-round activity.	We identified several periods of high influenza activity in Kenya throughout the year using defined criteria and modelled each period separately. As a result, more than one period of high influenza activity of a particular influenza type/subtype was modelled in some years in Kenya.<br><br>Because of significant year-round activity, the start and stop dates of each season were not easily ascertained and were selected based on the best fit of the model to the peak in activity. Dates of periods of high influenza activity may have started later than the true start date and ended earlier than the actual end date as these were not easily ascertainable.
Source of surveillance data	Influenza-like illness records from GP practices	Severe acute respiratory illness records from hospitalised patients	Ascertainment probability adjusted to reflect the probability of a hospitalised patient being detected. As a result, fitted model to surveillance data in Kenya has much lower numbers than that in the United Kingdom.
Age groups	0–4, 5–14, 15–44, 45–64, 65+ years	0-1, 1-5, 6-14, 15-19, 20-49, 50+ years	Kenya age groups were informed by the age groupings in the local social contact survey, the age-specific distribution of burden of illness, and the demographic pyramid in Kenya. Findings for the elderly age group are not as well defined in Kenya as compared to the United Kingdom because the ascertainment rate is much lower in this group.
Risk groups	Population stratified into high risk groups based on age and pre-existing conditions	No high-risk groups were included due to insufficient data on type and prevalence of influenza high-risk groups relevant in Kenya	Lack of stratification into high-risk groups in Kenya could lead to an underestimation of overall severe disease outcomes, which would be higher in high risk groups.

8 References

1. GeoData Institute. WorldPop datasets: University of Southampton; 2017 [cited 2017 August 3]. Available from: http://www.worldpop.org.uk/data/.
2. Noor AM, Zurovac D, Hay SI, Ochola SA, Snow RW. Defining equity in physical access to clinical services using geographical information systems as part of malaria planning and monitoring in Kenya. Tropical medicine & international health : TM & IH. 2003;8(10):917-26.
3. World Bank. Population growth (annual %) 2016 [cited 2016 February 1]. Available from: http://data.worldbank.org/indicator/SP.POP.GROW.
4. Burton DC, Flannery B, Onyango B, Larson C, Alaii J, Zhang X, et al. Healthcare-seeking behaviour for common infectious disease-related illnesses in rural Kenya: a community-based house-to-house survey. Journal of health, population, and nutrition. 2011;29(1):61-70.
5. Baguelin M, Flasche S, Camacho A, Demiris N, Miller E, Edmunds WJ. Assessing optimal target populations for influenza vaccination programmes: an evidence synthesis and modelling study. PLoS Med. 2013;10.
6. van Leeuwen E, Klepac P, Thorrington D, Pebody R, Baguelin M. fluEvidenceSynthesis: An R package for evidence synthesis based analysis of epidemiological outbreaks. PLOS Computational Biology. 2017;13(11):e1005838.
7. Kiti MC, Kinyanjui TM, Koech DC, Munywoki PK, Medley GF, Nokes DJ. Quantifying age-related rates of social contact using diaries in a rural coastal population of Kenya. PLoS One. 2014;9(8):e104786.
8. Carrat F, Vergu E, Ferguson NM, Lemaitre M, Cauchemez S, Leach S. Time lines of infection and disease in human influenza: a review of volunteer challenge studies. Am J Epidemiol. 2008;167.
9. Feikin DR, Njenga MK, Bigogo G, Aura B, Gikunju S, Balish A, et al. Additional diagnostic yield of adding serology to PCR in diagnosing viral acute respiratory infections in Kenyan patients 5 years of age and older. Clinical and vaccine immunology : CVI. 2013;20(1):113-4.
10. Kissling E, Valenciano M, Cohen JM, Oroszi B, Barret A-S, Rizzo C, et al. I-MOVE multi-centre case control study 2010-11: overall and stratified estimates of influenza vaccine effectiveness in Europe. PloS one. 2011;6(11):e27622-e.
11. Centers for Disease Control and Prevention (CDC) National Center for Immunization and Respiratory Diseases (NCIRD). Seasonal Influenza Vaccine Effectiveness, 2011-2012 2018 [updated 7 September 2018; cited 2019 June 30]. Available from: https://www.cdc.gov/flu/vaccines-work/2011-2012.html?CDC_AA_refVal=https%3A%2F%2Fwww.cdc.gov%2Fflu%2Fprofessionals%2Fvaccination%2Feffectiveness-year%2F2011-2012.html.
12. Centers for Disease Control and Prevention (CDC) National Center for Immunization and Respiratory Diseases (NCIRD). Seasonal Influenza Vaccine Effectiveness, 2013-2014 2018 [updated 7 September 2018; cited 2019 June 30]. Available from: https://www.cdc.gov/flu/vaccines-work/2013-2014.html?CDC_AA_refVal=https%3A%2F%2Fwww.cdc.gov%2Fflu%2Fprofessionals%2Fvaccination%2Feffectiveness-year%2F2013-2014.html.
13. Centers for Disease Control and Prevention (CDC) National Center for Immunization and Respiratory Diseases (NCIRD). Seasonal Influenza Vaccine Effectiveness, 2015-2016 2018 [updated 12 September 2018; cited 2019 June 30]. Available from: https://www.cdc.gov/flu/professionals/vaccination/effectiveness-year/2015-2016.html.
14. Flannery B, Chung JR, Belongia EA, McLean HQ, Gaglani M, Murthy K, et al. Interim Estimates of 2017-18 Seasonal Influenza Vaccine Effectiveness - United States, February 2018. MMWR Morbidity and mortality weekly report. 2018;67(6):180-5.
15. Kotecha RS, Wadia UD, Jacoby P, Ryan AL, Blyth CC, Keil AD, et al. Immunogenicity and clinical effectiveness of the trivalent inactivated influenza vaccine in immunocompromised children undergoing treatment for cancer. Cancer Med. 2015;5(2):285-93.

16. Turner N, Pierse N, Bissielo A, Huang Q, Radke S, Baker M, et al. Effectiveness of seasonal trivalent inactivated influenza vaccine in preventing influenza hospitalisations and primary care visits in Auckland, New Zealand, in 2013. Euro surveillance : bulletin Europeen sur les maladies transmissibles = European communicable disease bulletin. 2014;19(34).
17. Sullivan SG, Chilver MB, Carville KS, Deng YM, Grant KA, Higgins G, et al. Low interim influenza vaccine effectiveness, Australia, 1 May to 24 September 2017. Euro surveillance : bulletin Europeen sur les maladies transmissibles = European communicable disease bulletin. 2017;22(43).
18. The World Bank. GDP per capita (current US$) 2019 [cited 2019 9 June]. Available from: https://data.worldbank.org/indicator/NY.GDP.PCAP.CD?locations=KE.
19. International Monetary Fund. 5. Report for Selected Countries and Subjects 2018 [cited 2019 June 30]. Available from: https://www.imf.org/external/pubs/ft/weo/2018/01/weodata/weorept.aspx?sy=2010&ey=2023&scsm=1&ssd=1&sort=country&ds=.&br=1&pr1.x=28&pr1.y=7&c=664&s=NGDP_R%2CNGDP_D%2CNGDPRPC%2CNGDPRPPPPC%2CNGDPPC%2CNGDPDPC%2CPPPPC%2CPPPSH&grp=0&a=.
20. Central Bank of Kenya. Foreign Exchange Rates 2017 [updated 2017; cited 2019 June 30]. Available from: https://www.centralbank.go.ke/rates/forex-exchange-rates/.
21. Emukule GO, Ndegwa LK, Washington ML, Paget JW, Duque J, Chaves SS, et al. The cost of influenza-associated hospitalizations and outpatient visits in Kenya. BMC Public Health. 2019;19(3):471.
22. Mvundura M, Lorenson K, Chweya A, Kigadye R, Bartholomew K, Makame M, et al. Estimating the costs of the vaccine supply chain and service delivery for selected districts in Kenya and Tanzania. Vaccine. 2015;33(23):2697-703.
23. World Health Organisation. Global Health Observatory data repository 2018 [cited 2019 June 30]. Available from: http://apps.who.int/gho/data/?theme=main&vid=60850.
24. Institute for Health Metrics and Evaluation (IHME). Global Burden of Disease Study 2016 (GBD 2016) Disability Weights 2019 [cited 2019 June 9]. Available from: http://ghdx.healthdata.org/record/ihme-data/gbd-2016-disability-weights.
