# Supplementary figures and images for: Seasonal influenza vaccination in Kenya: an economic evaluation using dynamic transmission modelling
Source: BMC Med. 2020 Aug 20;18:223. doi: 10.1186/s12916-020-01687-7 (PMC7438179; doi:10.1186/s12916-020-01687-7)

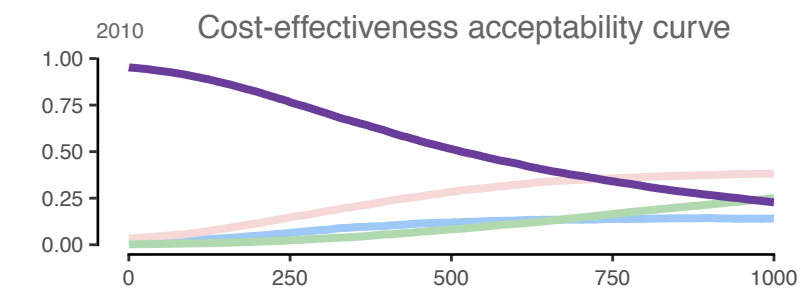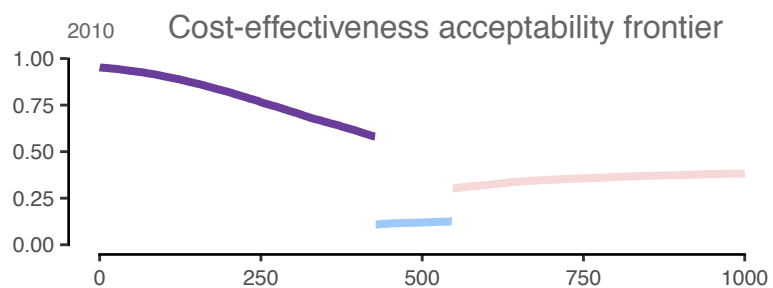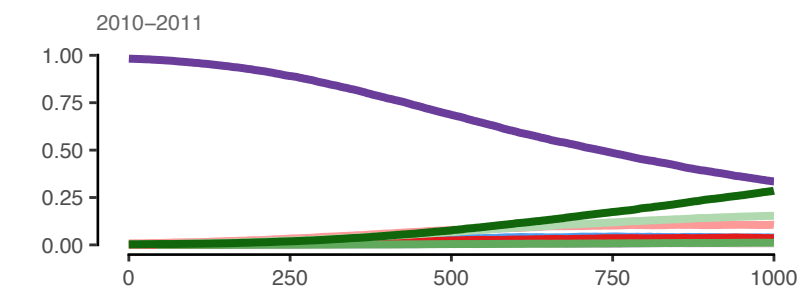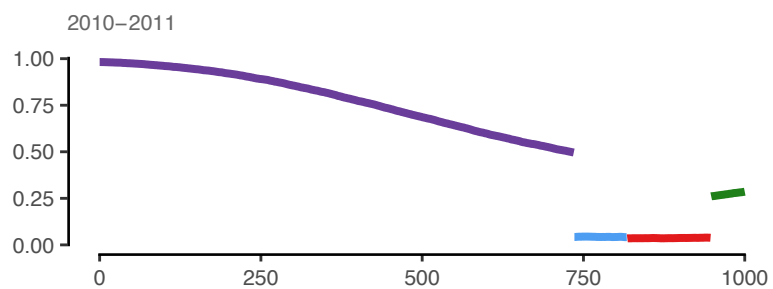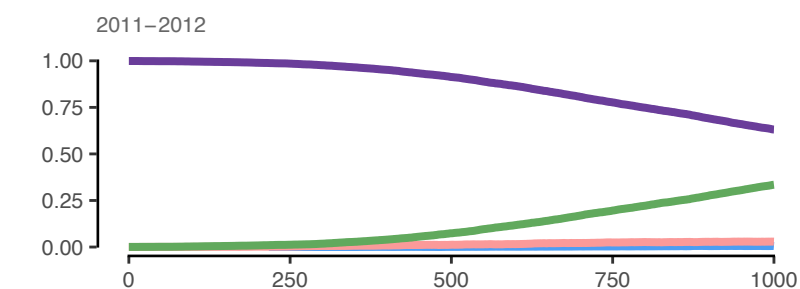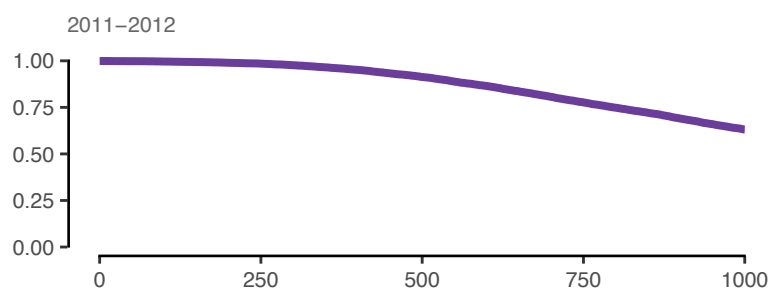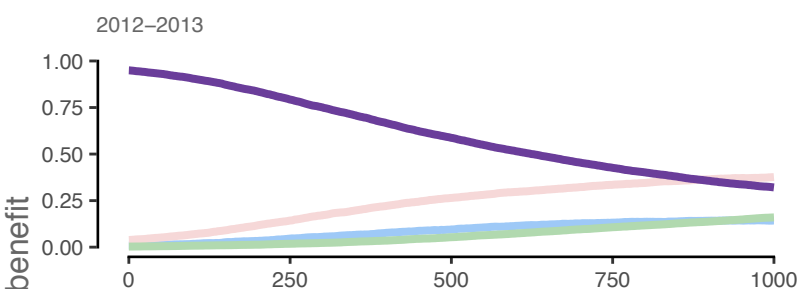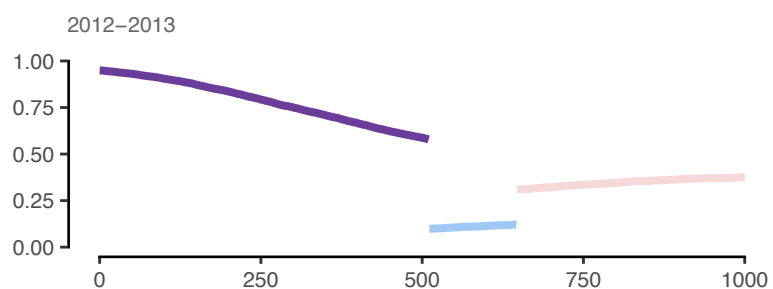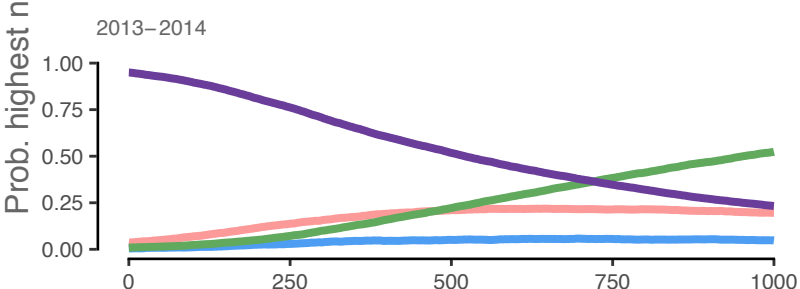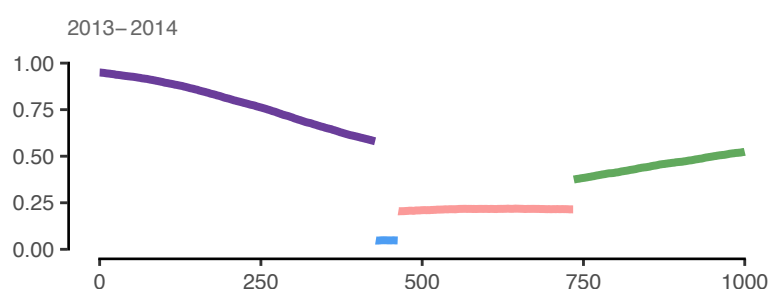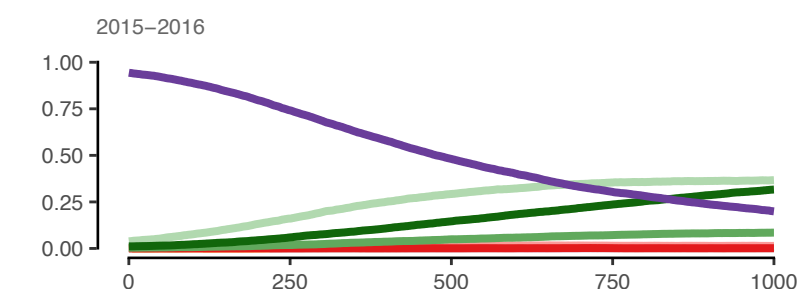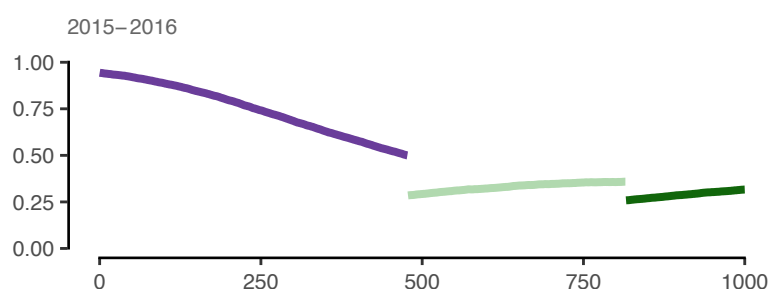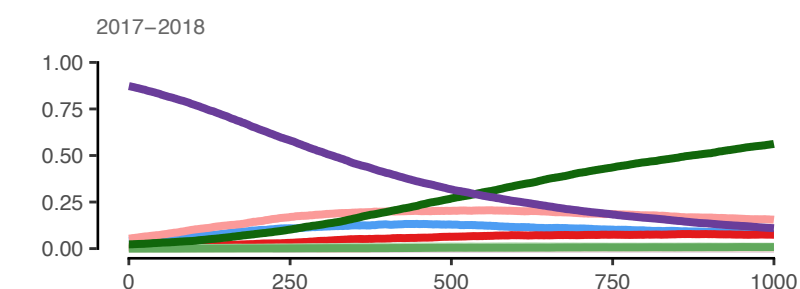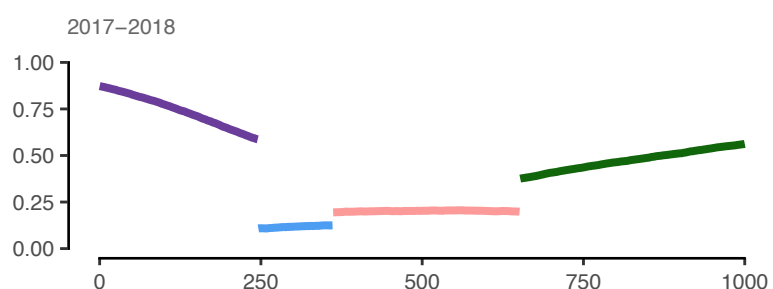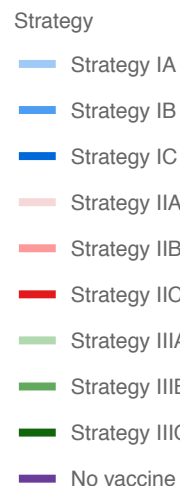

Supplement: Supplementary file 4 — Additional file 4. Yearly cost-effectiveness acceptability curves and frontiers for strategies with the highest incremental net monetary benefit considering total societal costs. NB: X axis is limited to 1000 USD per DALY averted. Strategies are vaccinating children 6–23 months (strategy I), 2–5 years (strategy II) and 6–14 years (strategy III) with either the SH influenza vaccine (Strategy A) or NH vaccine (Strategy B) or both (Strategy C: twice yearly 3-month vaccination periods, or Strategy D: year-round vaccination). [file 12916_2020_1687_MOESM4_ESM.pdf]

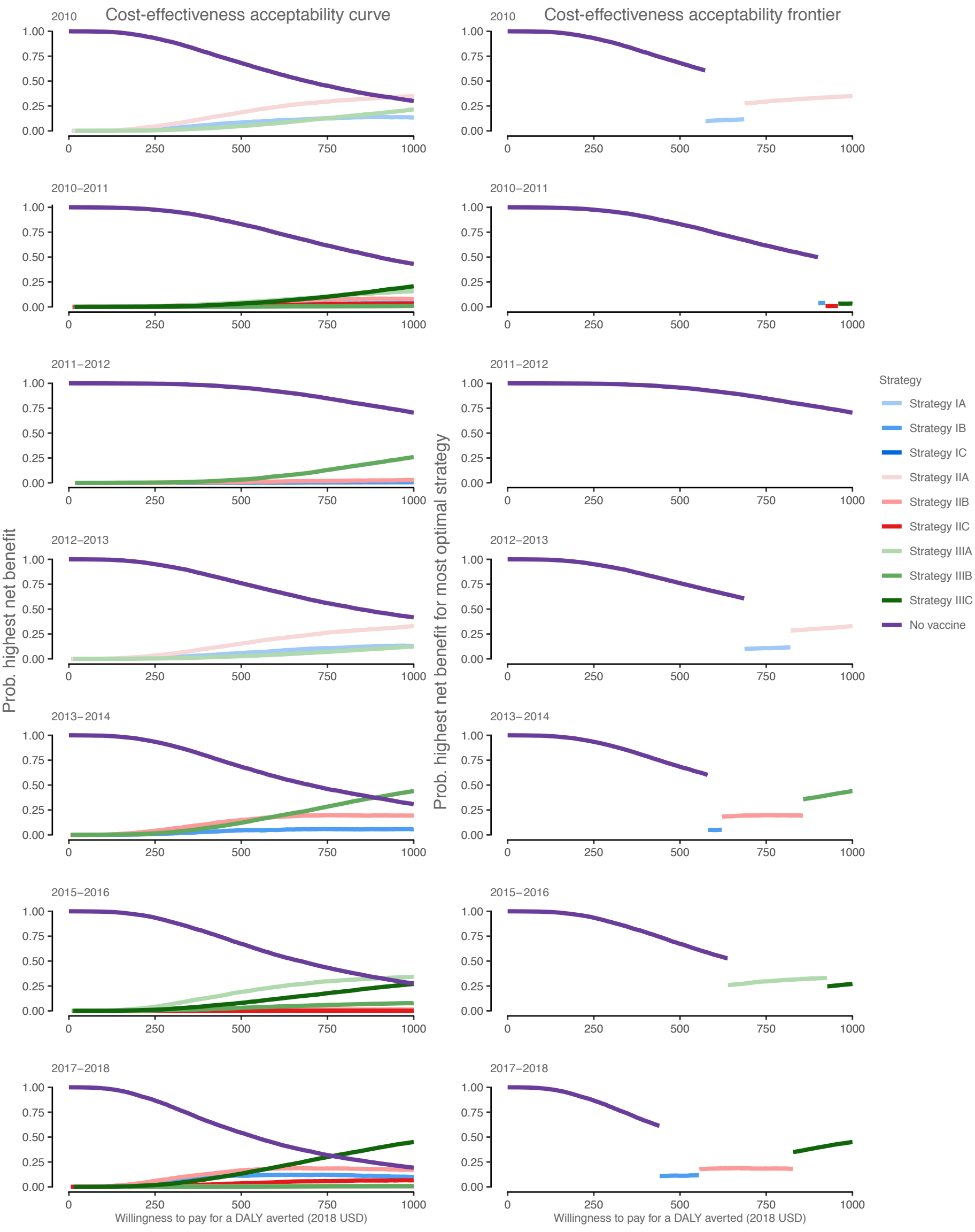

Supplement: Supplementary file 5 — Additional file 5. Yearly cost-effectiveness acceptability curves and frontiers for strategies with the highest incremental net monetary benefit considering direct medical costs only. NB: X axis is limited to 1000 USD per DALY averted. Strategies are vaccinating children 6–23 months (strategy I), 2–5 years (strategy II) and 6–14 years (strategy III) with either the SH influenza vaccine (Strategy A) or NH vaccine (Strategy B) or both (Strategy C: twice yearly 3-month vaccination periods, or Strategy D: year-round vaccination). [file 12916_2020_1687_MOESM5_ESM.pdf]
